# Supplementary material for: Prospective longitudinal study of ‘Sleepless in Lockdown’: unpacking differences in sleep loss during the coronavirus pandemic in the UK
Source: BMJ Open. 2022 Jan 3;12(1):e053094. doi: 10.1136/bmjopen-2021-053094 (PMC8724580; doi:10.1136/bmjopen-2021-053094)
Supplement: Supplementary data [file bmjopen-2021-053094supp001.pdf]

Appendix Table A. Sleep loss comparison before and after the COVID-19 pandemic by sex and ethnicity (N=8,163).

	Sleep loss before the COVID-19 pandemic (2019)	Sleep loss after the COVID-19 pandemic (April 2020)
Total	13.9	22.0
Sex	P<0.001	P<0.001
Men	10.2	13.5
Women	16.5	28.0
Ethnicity	P=0.05	P=0.438
British/English/Scottish/Welsh/Northern Irish (White)	13.5	21.7
Other White	15.5	23.3
Black, Asian, and Minority Ethnic (BAME)	17.2	24.3

Source: authors' analysis, Wave 1 of Understanding Society: COVID-19 Study, 2020.

Note: All proportions and number of respondents are unweighted. Pearson Chi-Square test applied.

Appendix Table B. Characteristics of participants, by ethnicity (N=8,163).

	British/English/Scottish/Welsh/Northern Irish (White)	Other White	Black, Asian, and Minority Ethnic (BAME)
Age group **% (n)**	P<0.001		
16-24	4.6 (199)	1.0 (1)	12.2 (54)
25-44	21.1 (1,286)	26.1 (76)	30.4 (194)
45-64	41.5 (3,114)	50.2 (174)	43.7 (268)
65-74	22.7 (1,850)	16.7 (72)	10.4 (48)
75+	10.1 (689)	5.9 (20)	3.3 (13)
Gender **% (n)**	P=0.111		
Men	46.2 (3,009)	38.6 (122)	47.0 (244)
Women	53.8 (4,129)	61.4 (221)	53.0 (333)
Subjective current financial situation **% (n)**	P<0.001		
Living comfortably	36.8 (2,949)	33.7 (127)	21.9 (149)
Doing alright	42.5 (3,016)	42.1 (147)	40.4 (228)
Just about getting by	16.1 (915)	19.3 (54)	28.1 (140)
Finding it quite difficult/very difficult	4.6 (258)	5.0 (15)	9.6 (60)
Subjective future financial situation **% (n)**	P<0.001		
About the same	79.3 (5,640)	66.8 (249)	68.9 (420)
Better off	5.8 (450)	10.4 (28)	6.7 (37)
Worse off	14.9 (1,048)	22.8 (66)	24.4 (120)
Children in the house **% (n)**	P<0.001		
No school aged child	76.3 (5,503)	68.5 (239)	60.0 (314)
At least one child aged 0-4	6.3 (449)	5.4 (24)	11.5 (72)
At least one school children aged 5-18	17.3 (1,186)	26.1 (80)	28.5 (191)
Key worker **% (n)**	P<0.001		
No	30.4 (2,130)	35.1 (121)	29.6 (189)
Yes	24.9 (1,709)	30.2 (87)	28.1 (177)
Not in paid or self-employed work	44.7 (3,299)	34.7 (135)	42.2 (211)
Has had symptoms that could be coronavirus **% (n)**	P=0.012		
No	89.8 (6,375)	90.6 (308)	83.7 (489)
Yes	10.2 (763)	9.4 (35)	16.3 (88)
Feel lonely **% (n)**	P<0.001		
Hardly ever	65.5 (4,889)	64.4 (228)	58.3 (329)
Sometime	27.0 (1,821)	26.2 (94)	33.2 (203)
Often	7.4 (428)	9.4 (21)	8.5 (45)
Prior sleep loss **% (n)**	P=0.557		
No	84.9 (6,176)	82.7 (290)	87.0 (478)
Yes	15.1 (962)	17.3 (53)	13.0 (99)
Region **% (n)**	P<0.001		
North East (ref)	5.0 (278)	1.5 (4)	0.7 (4)
North West	10.3 (684)	5.9 (20)	8.9 (62)
Yorkshire and The Humber	9.4 (633)	5.4 (19)	7.8 (54)
East Midlands	8.5 (611)	4.5 (14)	4.4 (30)
West Midlands	8.8 (563)	3.5 (21)	10.0 (76)
East of England	10.5 (725)	9.9 (28)	4.4 (41)
London	7.1 (373)	21.8 (65)	40.4 (210)
South East	15.7 (1,094)	12.4 (46)	11.5 (58)
South West	11.4 (820)	9.4 (30)	6.7 (20)
Wales	4.4 (423)	5.0 (16)	1.9 (12)
Scotland	7.5 (693)	5.4 (15)	3.3 (8)
Northern Ireland	1.6 (241)	15.3 (65)	0.1 (2)

Source: authors' analysis, Understanding Society: COVID-19 Study, 2020.

Note: All proportions are weighted using sample weights. The percentages are 100% within each ethnic group. Number of respondents are unweighted. Pearson Chi-Square test applied.

Appendix Table C. Sensitivity analyses: odds ratios and 95% CI of mixed-effects ordered logistic regressions.

	Model A All respondents			
	A1	A2	A3	A4
Gender				
Men (ref)				
Women	**3.6*** (3.2-4.0)**		**2.5*** (2.2-2.7)**	**3.4*** (3.0-3.9)**
Ethnicity				
British/English/Scottish/Welsh/Northern Irish (White) (ref)				
Other White		**1.6** (1.2-2.1)**	1.1 (0.9-1.4)	0.9 (0.7-1.2)
Black, Asian, and Minority Ethnic (BAME)		**1.6*** (1.3-2.1)**	0.9 (0.7-1.1)	**0.8* (0.6-0.9)**
Month				
April (ref)				
May			0.9 (0.9-1.0)	**1.1* (1.0-1.3)**
June			**1.1** (1.0-1.2)**	**1.4*** (1.3-1.6)**
July			**0.7*** (0.7-0.8)**	**0.8*** (0.5-0.6)**
September			**0.9 (0.9-1.0)**	**1.2** (1.1-1.4)**
November			**1.2*** (1.1-1.3)**	**1.5*** (1.3-1.6)**
January			1.1 (0.9-1.1)	**1.3*** (1.2-1.5)**
Interaction gender#month				
Women#May				**0.7*** (0.6-0.8)**
Women#June				**0.6*** (0.5-0.7)**
Women#July				**0.7*** (0.6-0.8)**
Women#September				**0.7*** (0.6-0.8)**
Women#November				**0.7*** (0.6-0.8)**
Women#January				**0.7*** (0.6-0.8)**
Interaction ethnicity#month				
BAME#May				**1.3* (1.0-1.7)**
BAME#June				1.2 (0.9-1.5)
BAME#July				**1.3* (1.0-1.7)**
BAME#September				0.9 (0.7-1.2)
BAME#November				1.1 (0.8-1.4)
BAME#January				1.2 (0.9-1.6)
Other White#May				1.3 (0.9-1.8)
Other White#June				1.2 (0.9-1.7)
Other White#July				1.0 (0.7-1.5)
Other White#September				1.2 (0.9-1.8)
Other White#November				1.2 (0.9-1.8)
Other White#January				1.1 (0.8-1.6)
LR test vs. ologit model (P value)	*<0.001*	*<0.001*	*<0.001*	*<0.001*
Number of person-month	*57,141*	*57,141*	*57,141*	*57,141*

Source: authors' analysis, Understanding Society: COVID-19 Study, 2020.

***p<0.001, **p<0.01, *p<0.05

Notes: Model A1 and A2 were bivariate models with gender or ethnicity in the model. Model A3 and A4 were multivariate models, controlling for age, highest qualification, subjective current financial situation, subjective future financial situation, live with a partner, children in the house, key worker, felt lonely, prior problem of sleep, region and month.

Appendix Figure 1. Prevalence of reported sleep loss pre and during the pandemic among analytical sample in Understanding Society mainstage survey in 2019 and Understanding Society: COVID-19 Study Wave 1 to Wave 7.

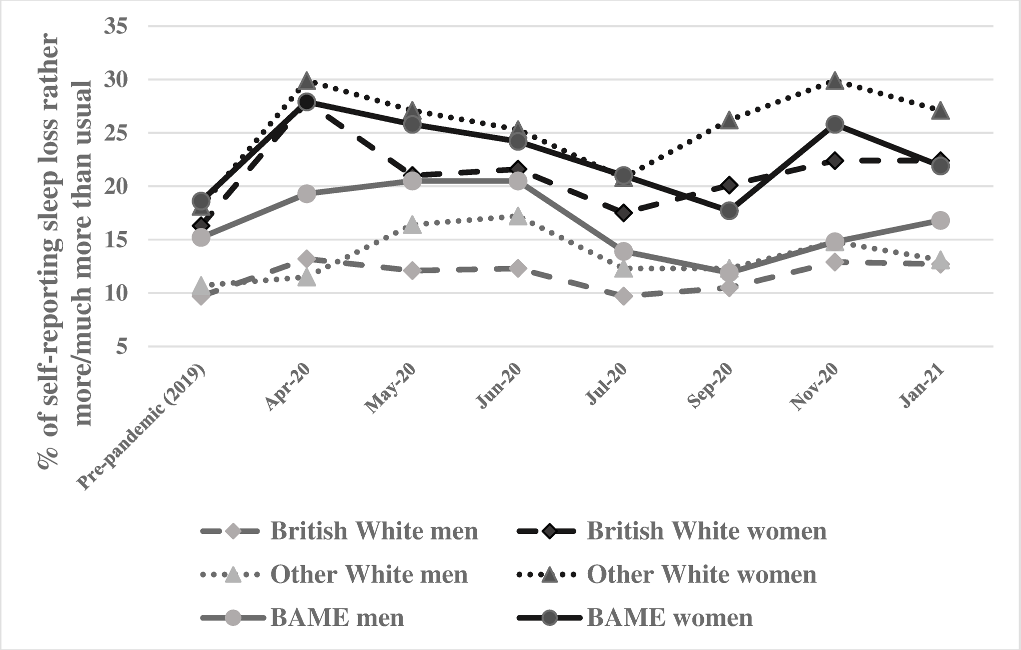

Source: authors' analysis, Understanding Society: COVID-19 Study, 2020.
Note: All proportions are unweighted. Black lines represent women in different ethnicities and grey lines represent men.
Number of respondents: N=8,163.
