# Supplementary material for: Prospective longitudinal study of ‘Sleepless in Lockdown’: unpacking differences in sleep loss during the coronavirus pandemic in the UK
Source: BMJ Open. 2022 Jan 3;12(1):e053094. doi: 10.1136/bmjopen-2021-053094 (PMC8724580; doi:10.1136/bmjopen-2021-053094)
Supplement: Author's manuscript [file bmjopen-2021-053094.draft_revisions.pdf]

BMJ Open is committed to open peer review. As part of this commitment we make the peer review history of every article we publish publicly available.

When an article is published we post the peer reviewers' comments and the authors' responses online. We also post the versions of the paper that were used during peer review. These are the versions that the peer review comments apply to.

The versions of the paper that follow are the versions that were submitted during the peer review process. They are not the versions of record or the final published versions. They should not be cited or distributed as the published version of this manuscript.

BMJ Open is an open access journal and the full, final, typeset and author-corrected version of record of the manuscript is available on our site with no access controls, subscription charges or pay-per-view fees (http://bmjopen.bmj.com).

If you have any questions on BMJ Open's open peer review process please email info.bmjopen@bmj.com

BMJ Open

A prospective longitudinal study of "Sleepless in Lockdown": unpacking differences in sleep loss during the coronavirus pandemic in the UK

Journal:	*BMJ Open*
Manuscript ID	bmjopen-2021-053094
Article Type:	Original research
Date Submitted by the Author:	05-May-2021
Complete List of Authors:	Falkingham, J; ESRC Centre for Population Change, Faculty of Social Sciences, University of Southampton<br>Evandrou , Maria ; University of Southampton Centre for Research on Ageing, Faculty of Social Sciences; ESRC Centre for Population Change, Faculty of Social Sciences, University of Southampton<br>Qin, Min; ESRC Centre for Population Change, Faculty of Social Sciences, University of Southampton<br>Vlachantoni, Athina; University of Southampton Centre for Research on Ageing, Faculty of Social Sciences; ESRC Centre for Population Change, Faculty of Social Sciences, University of Southampton
Keywords:	COVID-19, EPIDEMIOLOGY, MENTAL HEALTH

SCHOLARONE™
Manuscripts

BMJ

I, the Submitting Author has the right to grant and does grant on behalf of all authors of the Work (as defined in the below author licence), an exclusive licence and/or a non-exclusive licence for contributions from authors who are: i) UK Crown employees; ii) where BMJ has agreed a CC-BY licence shall apply, and/or iii) in accordance with the terms applicable for US Federal Government officers or employees acting as part of their official duties; on a worldwide, perpetual, irrevocable, royalty-free basis to BMJ Publishing Group Ltd ("BMJ") its licensees and where the relevant Journal is co-owned by BMJ to the co-owners of the Journal, to publish the Work in this journal and any other BMJ products and to exploit all rights, as set out in our licence.

The Submitting Author accepts and understands that any supply made under these terms is made by BMJ to the Submitting Author unless you are acting as an employee on behalf of your employer or a postgraduate student of an affiliated institution which is paying any applicable article publishing charge ("APC") for Open Access articles. Where the Submitting Author wishes to make the Work available on an Open Access basis (and intends to pay the relevant APC), the terms of reuse of such Open Access shall be governed by a Creative Commons licence – details of these licences and which Creative Commons licence will apply to this Work are set out in our licence referred to above.

Other than as permitted in any relevant BMJ Author's Self Archiving Policies, I confirm this Work has not been accepted for publication elsewhere, is not being considered for publication elsewhere and does not duplicate material already published. I confirm all authors consent to publication of this Work and authorise the granting of this licence.

For peer review only

A prospective longitudinal study of "Sleepless in Lockdown": unpacking differences in sleep loss during the coronavirus pandemic in the UK

Jane Falkingham, Maria Evandrou, Min Qin and Athina Vlachantoni

Authors' affiliations

Jane Falkingham (Corresponding Author)
ESRC Centre for Population Change, Faculty of Social Sciences, University of Southampton, Southampton, UK
j.c.falkingham@soton.ac.uk
07872423609

Maria Evandrou
Centre for Research on Ageing and ESRC Centre for Population Change, University of Southampton, Southampton, UK

Min Qin
ESRC Centre for Population Change, Faculty of Social Sciences, University of Southampton, Southampton, UK

Athina Vlachantoni
Centre for Research on Ageing and ESRC Centre for Population Change, University of Southampton, Southampton, UK

For peer review only

Abstract

Objectives: COVID-19 has disproportionate impact on Black, Asian, and Minority Ethnic (BAME) groups and women. Concern over direct and indirect effects may also impact upon sleep. We explore the levels and social determinants of self-reported sleep loss among the UK population during the pandemic, focusing on ethnic and gender disparities.
Setting: This prospective longitudinal study analysed data from seven waves of the Understanding Society (USoc) COVID-19 Study collected from April 2020 to January 2021 linked to 2019 pre-pandemic data from the mainstage interviews, providing baseline information about the respondents prior to the pandemic.
Participants: The analytical sample included 8,163 respondents aged 16 and above who took part in all seven waves with full information on sleep loss, defined as experiencing 'rather more' or 'much more' than usual sleep loss due to worry, providing 57,141 observations of data.
Primary outcome measures: Self-reported sleep loss. Mixed-effects regression models were fitted to consider within- and between-individual differences.
Results: Women were more likely to report sleep loss than men (odds ratio [OR] 2.1 [95% CI 1.9–2.4]) over the ten-month period. Being female, having young children, perceived financial difficulties and COVID-19 symptoms were all predictive of sleep loss. Once these covariates were controlled for, the bivariate relationship between ethnicity and sleep loss (1.4 [1.6–2.4]) was reversed (0.7 [0.5–0.8]). Moreover, the strength of the association between gender and ethnicity and the risk of sleep loss varied over time, being weaker among women in July (0.6 [0.5–0.7]), September (0.7 [0.6–0.8]), November (0.8 [0.7–1.0]) and January (0.8 [0.7–0.9]) compared with April, but positively stronger among BAME individuals in May (1.4 [1.0–2.1]), weaker only in September (0.7 [0.5–1.0]).
Conclusions: The pandemic has widened sleep deprivation disparities, with women with young children, COVID-19 infection and BAME individuals experiencing sleep loss, which may adversely affect their mental and physical health.

Strengths and limitations of this study

- This is a large-scale community-based prospective longitudinal study.
- We examined the trends and patterns of sleep problems and the impact of known correlates of being infected with, or affected by, COVID-19 including ethnicity and gender.
- Sleep loss was measured by self-reports. As no definition of sleep loss was provided, participants may have used different definitions while answering the question.
- Attrition could have confounded the interested prospective associations.

For peer review only

For peer review only - http://bmjopen.bmj.com/site/about/guidelines.xhtml

Funding: This research was supported by the Economic and Social Research Council Centre for Population Change (grant number ES/K007394/1) at the University of Southampton.

Keywords: COVID-19; Pandemic; Sleep Loss; Mental Health; Gender; Ethnicity

Competing Interest Statement
The authors have declared no competing interest.

Ethics approval
This study uses secondary data for the collection of which ethical approval has been obtained by the survey team. All relevant ethical guidelines at the University of Southampton have been followed.

Authors' contributions
All authors (JF, ME, MQ, AV) contributed equally to the initial discussion of the idea behind the manuscript, to the design of the manuscript, to the analysis plan and to the redrafting and finalisation of the manuscript. MQ conducted the statistical analysis for the paper and drafted sections of the initial draft of the manuscript.

Role of the funding source
This research was supported by the Economic and Social Research Council Centre for Population Change (grant number ES/K007394/1) at the University of Southampton. This grant paid for the staff time dedicated to the discussion around the manuscript and the writing of the manuscript. None of the authors has been paid to write this article by a pharmaceutical company or other agency. JF who is the corresponding author had full access to all the data in the study and had final responsibility for the decision to submit the paper for publication.

No additional data available.

For peer review only

For peer review only - http://bmjopen.bmj.com/site/about/guidelines.xhtml

A prospective longitudinal study of “Sleepless in Lockdown”: unpacking differences in sleep loss during the coronavirus pandemic in the UK

Introduction

The coronavirus disease (COVID-19) is impacting upon physical and mental health globally. Sleep problems associated with increased psychosocial stressors induced by the coronavirus itself, and as a result of the social distancing measures that have been introduced to manage the virus, are emerging as a significant outcome of the COVID-19 crisis. According to a report last summer, more than half of the UK population has struggled with sleep during the first lockdown [1]. Sleep has long been recognised as an essential determinant of human health and performance. Good sleep restores energy, promotes healing, interacts with the immune system and impacts upon behaviour [2]. Even acute sleep deprivation can impair judgement and cognitive performance, while persistent deviations have been linked to disease development and increased mortality [3, 4]. During the pandemic, lack of sleep may itself have had knock-on effects on people's capacity to be resilient. However, to date relatively limited research has been conducted on sleep deprivation during the pandemic.

Sleep is known to be regulated by circadian rhythms, sleep-wake homoeostasis and cognitive-behavioural influences [3]. With regards to environmental, behavioural and health determinants, poor sleep has been associated with stress, anxiety, work pressures, financial concerns, mental and physical impairments, and physical activity [5, 6, 7, 8]. Previous studies have found that women were more likely than men to have trouble falling and staying asleep frequently, or to have insufficient sleep [9, 10]. The relationship between ethnicity and sleep is complicated due to the broader social and environmental factors determining group differences in sleep behaviours and the structural relationships between these factors and ethnicity [11]. Some studies reported that inadequate sleep duration and poorer sleep were more prevalent among low-income and Black, Asian and Minority Ethnic (BAME) communities [12], whereas others have failed to find this association [13].

In understanding the relationship between COVID-19 and sleep, it is helpful to conceptually distinguish between those factors linked to being *infected* with COVID-19 and those associated with the policy responses and measures introduced to manage the pandemic that have *affected* everyday life. Although it is still relatively early in our understanding of COVID-19, research by Public Health England 2020 has highlighted that older people, men and individuals from BAME groups are all at increased risk of developing a severe response to the virus and to die from it [14]. The reasons for the heightened risk amongst certain ethnic groups remains unresolved, but potential contributors include the disproportionate representation of BAME individuals in some high risk occupations including front line health care work, as well as wider environmental factors that, interwoven with issues of inequality, deprivation and structural racism, manifest in longstanding ethnic disparities in health [15]. A priori, we might expect those groups facing the greatest health risks from the virus to report increased sleep loss due to worry and thus to observe differences across ethnic groups.

The public health actions taken to control the spread of the virus have, however, impacted all domains of life and thus affected all individuals. On 23rd March 2020, the UK went into lockdown

For peer review only - http://bmjopen.bmj.com/site/about/guidelines.xhtml

in an unprecedented attempt to limit the spread of coronavirus, with the Government mandating all those who could to work at home, closing schools, restaurants and all but essential shops, and advising the population to stay at home and limit contact with other individuals outside their household. On 5th November, a second national lockdown came into force in England, followed by the third nationwide lockdown on 5th January 2021, the phased end of which si scheduled for 21 June. The resultant move to home working and learning and, for some, the loss of work altogether, along with limited social contact and increased isolation, may all be anticipated to affect mental well-being and the ability to sleep. Preliminary evidence points towards the young and women being disproportionally affected, with women being more likely than men to be working in sectors that were locked down [16] and mothers being more likely to be interrupted whilst working from home than fathers [17]. Lockdown has also resulted in increased instances of domestic violence; the UK domestic abuse organisation, Refuge, reported a 25 per cent increase in calls and online requests since the first lockdown began in March 2020 [18]. Given this, we might anticipate a gender differential in increased sleep loss, with women being disproportionately affected by lockdown compared with men.

This study aims to provide novel evidence regarding patterns of self-reported increased sleep loss due to worry during the first four weeks of the COVID-19 related lockdown in the UK. Using recently collected nationally representative survey data, the research provides the first estimates of the prevalence and incidence of increased sleep loss since the coronavirus pandemic. It attempts to unpack the impact of factors associated with being *infected* and being *affected* by COVID-19, with a particular focus on the extent to which the pandemic has exacerbated differentials in sleep loss by ethnicity and gender.

Methods

Study design and population

We analysed data from the Understanding Society COVID-19 study [19] covering ten months and three COVID-19 related national lockdown in the UK. Data was collected online monthly. The first wave of the COVID-19 survey was fielded between 24th-30th April 2020, with Wave 2 taking place in May, Wave 3 in June, Wave 4 in July, Wave 5 in September, Wave 6 in November and Wave 7 in December 2020/January 2021. There was no survey in August or October. The UK Household Longitudinal Study [20] (UKHLS; Understanding Society) is an ongoing panel survey of more than 40,000 households that began in 2009. Between 24-30th April 2020, members of households who participated in either of the two most recent UKHLS data collections (Waves 8 or 9), and who were older than 16 years, were invited to complete the first wave of the COVID-19 web survey. The probability sample was drawn from postal addresses. Northern Ireland and areas in England, Scotland, and Wales with proportionately large migrant and ethnic minority populations were oversampled. All household members aged 16 or older in April 2020 were invited to participate, except for those unable to make an informed decision as a result of incapacity, and those with unknown postal addresses or addresses abroad. The response rate (full interview) of seven waves of the Understanding Society COVID-19 study was 39%, 35%, 33%, 32%, 30%, 28% and 28% respectively [19].

For peer review only - http://bmjopen.bmj.com/site/about/guidelines.xhtml

The Understanding Society COVID-19 study is funded by the Economic and Social Research Council and the Health Foundation with scientific leadership by the Institute for Social and Economic Research, University of Essex. Fieldwork for the survey is carried out by Ipsos MORI and Kantar. The research data are distributed by the UK Data Service.

17,452 respondents took part in Wave 1 of the COVID-19 study. The inclusion criteria of the analytical sample for this study were all respondents aged 16 and over, who had participated in all seven waves survey and had no missing values on the outcome variable, constituting a final sample size of 8,163 individuals and 57,141 observations. The characteristics of the analytical sample are shown in Table 1. The analytical sample was slightly older and wealthier than the baseline sample (Appendix Table A). Women were more likely to continue to participate in the follow-up studies, but BAME individuals were less likely. Loss to follow-up was slightly more likely amongst those who reported sleep loss at wave 1 of the COVID-19 study (Appendix Table A).

Procedures

The outcome variables included whether the respondent reported an increase in sleep loss over worry in the last few weeks. The question on sleep loss was identical across both the Understanding Society COVID-19 Study and the mainstage data in 2019. As participants were not provided with a definition of sleep loss, it is recognised that different participants may have used different definitions when answering the question. The specific question wording, along with the four response categories, is presented in the text box below.

The next questions are about how you have been feeling over the last few weeks.

Have you recently lost much sleep over worry?
1. Not at all
2. No more than usual
3. Rather more than usual
4. Much more than usual

Source: University of Essex (2021).

In addition to the general sleep loss question, in the Wave 4 COVID-19 study, participants were asked a more detailed set of questions about sleep including: How many hours of actual sleep did you usually get per night during the last month? How often have you had trouble sleeping because you cannot get to sleep within 30 minutes? How often have you had trouble sleeping because you wake up in the middle of the night or early in the morning? These more comprehensive measures were compared against the general sleep loss measure as part of the descriptive analyses.

For the purposes of the multi-variate analysis, a respondent was defined as experiencing sleep loss during the lockdown if he or she reported sleep loss over worry in the last few weeks 'rather more than usual' or 'much more than usual' in the Understanding Society COVID-19 Study. The outcome variables were binary (1=yes; 0=no).

A range of explanatory variables were included, reflecting both known associates of sleep loss [9, 21] as well as those that we hypothesise may be associated with heightened anxiety during the pandemic.

For peer review only - http://bmjopen.bmj.com/site/about/guidelines.xhtml

For peer review only

Demographic and socioeconomic characteristics included age, gender, ethnicity and educational qualification. Gender distinguished between men and women; the survey responses do not differentiate those whose reported sex has changed since birth or those who classify themselves as intersex. Ethnicity was classified into three groups: British/English/Scottish/Welsh/Northern Irish (White), Other White, and Black, Asian, and Minority Ethnic (BAME).

Variables capturing factors associated with COVID-19 itself included whether the respondent reported having experienced symptoms that could be coronavirus and being a key worker [1]. Other variables aimed to capture the impacts of the policy response to COVID-19, particularly the effect of lockdown. Increased stress related to childcare and home schooling was proxied by the presence of children in the house (whether at least one child aged 0-4 or school-aged child), and whether the respondent was living with a partner. Exposure to financial stress was proxied by two variables capturing the respondents subjective view of their current and future financial situation. Social isolation was measured by the frequency of having felt lonely in the last 4 weeks. In order to capture regional differences in the intensity of the pandemic [2], a variable reflecting the respondents' place of respondence was included, based on the UK government office region (North East, North West, Yorkshire and The Humber, East Midlands, West Midlands, East of England, London, South East, South West, Wales, Scotland, and Northern Ireland). Finally, in order to capture time effects, we controlled for the survey time point, measured as a dummy variable (April, May, June, July, September, November and December/January).

Sleep loss and certain covariates such as the frequency of feelings of loneliness in the previous four weeks, has had symptoms that could be coronavirus, young children present in the household, and the respondents' perceived current and future financial situation, were measured at each wave of COVID-19 study. For some individuals, their responses to these questions changed over time during the follow-up period; thus, in the statistical models these were all treated as time-varying variables. Other covariates were treated as time-invariant variables. It is recognised that pregnancy, menstrual cycle and menopause may all be linked to sleep. Data on these was not available. However, age was included as a co-variant, which will partially capture whether women have reached the menopause.

Analytical approach

Descriptive statistics illustrating the prevalence of reported sleep loss (stratified by gender and ethnicity) pre and during the pandemic among *all* participants aged 16 and above in the USoc mainstage survey in 2019 and in the COVID-19 Study Waves 1 to 7 were calculated (Figure 1).

Subsequent analysis was then restricted to the analytical sample described above. Mixed-effects logistic models were used to assess the existence and strength of associations between sleep loss and COVID-19-related circumstances during the pandemic. Given that the data collected from an

[1] According to Department of Health and Social Care guidance on testing eligibility (2020) [22], key workers are people whose jobs are vital to public health and safety during the coronavirus lockdown. The list includes health and social care, e.g. all NHS staff, frontline health and social care staff such as doctors, nurses, plus support and specialist staff in the health and social care sector; education and childcare, including social workers; food and other necessary goods; key public services; local and national government; utility workers; public safety and national security; and transport.
[2] In the first few weeks of the pandemic in the UK, the rate of infection was much higher in London than elsewhere in the country.

For peer review only - http://bmjopen.bmj.com/site/about/guidelines.xhtml

individual over ten months are not independent of each other, mixed-effects models are the recommended statistical technique to take into consideration between-individuals variance and within-individuals variance [23]. This method has the advantage of including both fixed and random effects. The former are model components used to define systematic relationships such as overall changes of sleep loss over time and/or social determinants induced individual differences, and the latter account for the variability among individuals around the systematic relationships captured by the fixed effects.

For this study, the logistic mixed-effects models included sleep loss as the response variable, and gender, ethnicity group and timepoint as fixed effects. Observations by person-month were attached to each respondent; thus, in order to estimate the random intercepts, we accounted for random variation between individuals and between observations within the same individual. We modelled an interaction between time and gender, as well as between time and ethnicity, as a fixed effect to examine whether the association between gender and ethnicity varied according to time. Other potential confounding variables included the respondents' age, highest qualification, subjective current financial situation, subjective future financial situation, whether they were living with a partner, any children in the house, whether they were a key worker, the frequency of feelings of loneliness in the previous four weeks, any prior problem of sleep, and region. Regression analyses were carried out for the total population and then for men and women separately. Results were presented as odds ratios of sleep loss by gender and ethnicity, with 95% CIs and associated two-sided p values.

A sensitivity analysis was conducted in order to check the robustness of the results. Here we measured sleep loss as an ordered variable with four categories (not at all, no more than usual, rather more than usual and much more than usual) instead of two. Mixed effects ordered logistic regression was applied. The analyses were carried out in STATA version 15 [24].

Patient involvement

No patients were involved in this study.

Results

Descriptive analyses

Figure 1 shows the level of reported sleep loss by gender and ethnicity among all participants aged 16 and above in the USoc mainstage survey in 2019 and in the USoc: COVID-19 Study Wave 1 to Wave 7. The first data point provides a baseline, pre-pandemic, with subsequent data points then illustrating how sleep loss changed during the pandemic, with the first two national lockdowns highlighted, the third starting in January 2021. Black lines represent women in different ethnicities and grey lines represent men. Overall, women reported much higher levels of sleep loss than men; and respondents from ethnic minority groups had a higher prevalence of sleep loss than British White respondents among both men and women, with BAME women experiencing the highest prevalence. Differentials in the prevalence of reporting sleep loss by gender and ethnicity are evident at baseline; however, the gap appears to have widened during the pandemic and was especially pronounced in April 2020, illustrating the effect of the early stages of the pandemic and the first lockdown, when the prevalence of sleep loss rose amongst all groups, with the rise particularly

For peer review only - http://bmjopen.bmj.com/site/about/guidelines.xhtml

marked amongst all women and BAME men. As the first lockdown eased, the prevalence of sleep loss fell, reaching a low in July / September, although for most groups this remained slightly above the pre pandemic baseline. Sleep loss then increased during the autumn, with a second peak coinciding with the second national lockdown in November 2020.

Restricting the analytical sample to all those with complete information across all seven waves, Table 2 shows that one in four people in the UK reported increased sleep loss due to worry during the first four weeks of the first coronavirus pandemic lockdown in spring 2020 (Table 2). There were clear differences between women and men and across ethnic communities. Strikingly, although men have been found to face a higher risk of experiencing severe symptoms and dying from COVID-19, women were twice as likely as men to report that they had lost sleep 'much more than usual' (5.8% v 1.9%) and 'rather more than usual' (23.9% v 11.8%), supporting the hypothesis that women were disproportionately affected by the economic and social consequences of the first lockdown. In the consecutive months, the proportion reporting increased sleep loss slightly decreased amongst both men and women, but women still showed a much higher proportion of increased sleep loss than men.

The results by ethnicity do, however, support the argument that the risk of infection may play a role; in the first month of lockdown in spring 2020, 6.3% of BAME respondents reported being 'much more than usual' to have lost sleep through worry over the last few weeks compared to 3.7% of white respondents (Table 2).

The prevalence of sleep loss ('rather more than usual' and 'much more than usual') in April 2020 (22.0%) was higher than that reported prior to the pandemic (13.9%) and the differentials between men and women, and individuals from different ethnic communities have widened during the epidemic (Appendix Table B).

Looking at the more detailed information available in Wave 4 (July 2020) shows that on average, those who reported problems with sleep slept around 45 minutes less than those who did not have trouble sleeping (Table 3). The majority of respondents reporting sleep loss had trouble getting to sleep within 30 minutes or reported waking up in the middle of the night or early in the morning. They were also more likely to rate their overall quality of sleep fairly bad or very bad. They were also more likely to report having trouble sleeping because of having a cough or snoring loudly, taking medicine to help them sleep, and having trouble staying awake while driving, eating means, or engaging in social activity (Table 3), indicating that the general sleep loss question is a good proxy for these wider set of sleep related issues.

Multi-variate analysis

Many of the characteristics associated with sleep loss are likely to be interrelated; for example, those with a child aged 0-4 at home are also likely to be aged 25-44; those who are key workers are more likely to have experienced symptoms etc. To further unravel the picture, a series of multivariate logistic regression models were run; firstly, for the population as a whole, and then separately for men and women. The first two columns (A1 and A2) for Model A (Table 4) show the unadjusted (bi-variate) odds ratios of experiencing sleep loss by gender and ethnicity (OR=3.2, 95% CI=2.8–3.6; OR=1.4, 95% CI=1.6–2.4), whilst the third column (A3) shows the adjusted odds of the main effect for the full model.

For peer review only - http://bmjopen.bmj.com/site/about/guidelines.xhtml

The analysis shows that the differential reporting of sleep loss by gender remains significant even after controlling for all other factors (OR=2.1, 95% CI=1.9–2.4); the differential by ethnicity is however reversed (OR=0.7, 95% 0·5–0.8) once other factors are taken into account such as being a key worker, having had symptoms, having children in the household, experiencing financial difficulties and living in London, the initial epicentre of the UK's COVID-19 outbreak in spring 2020. Individuals from BAME communities are disproportionately represented in all of these groups [3] (Appendix Table C). By including interactions between gender and ethnicity with month, Model A4 assessed the effects of gender and ethnicity on shifts over time. The results show that the strength of the association between gender and ethnicity and the risk of sleep loss varied over time. Women presented a lower risk of sleep loss in May, June, July, November and January 2021 as compared to the early stages of the first lockdown in April 2020 (OR = 0.7, 95 % CI = 0.5 – 0.8; OR=0.6, 95% CI=0.5–0.8; OR=0.7, 95% CI=0.6–0.9; OR=0.8, 95% CI=0.6–0.9; OR=0.7, 95% CI=0.6–0.9). By contrast, the BAME group experienced a higher risk of sleep loss in May (OR=1.4, 95% CI=1.0–2.1), and the risk only started to fall in September (0.7 [0.5–1.0]).

The analysis shows that the coronavirus infection, with school-aged children at home, feeling lonely, perceived financial difficulties and worry, being a woman, and month were all predictive factors of sleep loss (Table 4). The influential factors were slightly different among men (Model B) and women (Model C). Among women, experiencing coronavirus symptoms was a risk factor, whilst being of BAME heritage reduced the risk of increased sleep loss *once other factors were controlled for*, whilst both these factors were not significant for men.

In the sensitivity analysis where sleep loss was measured as an ordered variable, the analysis of the data with mixed-effect ordered logistic regression rather than mixed-effect logistic regression did not change the pattern of results (Appendix Table D).

Discussion and conclusion

This study has revealed several important findings related to sleep health during the COVID-19 pandemic. Firstly it provides robust evidence that sleep loss is affecting more people during the COVID-19 pandemic than previously, reflecting the fact that stress levels have risen due to anxieties about health, financial consequences, changes in social life and the daily routine, all of which may affect sleep homeostasis.

The study also provides evidence that women have been more vulnerable to sleep deprivation during lockdown, which is in line with previous research suggesting that women have more sleep disturbances than men [9,21], and that women are more prone to stress-related sleep disorders such as post-traumatic stress disorder and anxiety disorders [25]. There is emerging evidence that mental health experiences during the COVID-19 pandemic in the UK differ between men and women, with more women suffering from anxiety in the early stages of lockdown [26]. Women's position in the labour market may increase their exposure to COVID-19, as women represent a significant majority of frontline workers in social care, education and health care [16, 27]. Many parents will have been affected

[3] A range of interaction effects with ethnicity were investigated but none were significant and thus are not included in the final model.

For peer review only - http://bmjopen.bmj.com/site/about/guidelines.xhtml

by school closures, and requirements to balance paid work with increasing childcare and providing support to their children's learning. However, the gendered allocation of childcare means that in many households, it is the mother who has continued to provide the majority of primary care for children. Furthermore, many mid-life women find themselves juggling employment with caring responsibilities for aged parents and grandchildren [28].

Individuals from BAME communities showed a higher prevalence and incidence rate of sleep loss than British White individuals. This reflects the fact that those of BAME heritage have disproportionally higher rates of coronavirus infection, high anxiety associated with coronavirus-specific circumstances [22], are more likely to be key workers, to have dependent children, and to feel lonely. All of these factors are likely to increase the risk of sleep loss, with the result that once all these other factors are controlled for, being a member of a BAME community was associated with a *reduced* chance of sleep loss – highlighting the complex relationship between ethnicity and sleep health.

Some limitations should be acknowledged. First, sleep loss was measured by self-reports and is therefore subject to recall bias and participants' perceptions [29]. Secondly, attrition between data collection waves and missing values on the outcome variable meant that follow-up data were not available for more than half of participants who were surveyed in Wave 1. The sub-sample lost to follow-up were older and less affluent than those who participated in subsequent waves. This attrition could have confounded the identification of prospective associations between gender, ethnicity and sleep loss, in that more vulnerable people were not retained in the analyses.

In conclusion, the COVID-19 pandemic and the policy responses to it, including home working and schooling, have widened the disparity of sleep deprivation across gender and ethnicity, putting women and ethnic minorities at an even greater disadvantage. Disrupted and poor sleep is associated with wider mental and physical health challenges. Policy makers and health professionals need to take action now to support and promote better sleep health amongst vulnerable groups during the pandemic, averting future secondary complications.

For peer review only

For peer review only - http://bmjopen.bmj.com/site/about/guidelines.xhtml

Table 1. Sample characteristics (Wave1).

	%	Number of respondents
Total	100.0	8,163
Age group		
16-24	4.8	309
25-44	22.0	1,587
45-64	41.7	3,571
65-74	21.8	1,972
75+	9.6	724
Gender		
Men	46.1	3,418
Women	53.9	4,745
Ethnicity		
British/English/Scottish/Welsh/Northern Irish (White)	91.5	7,138
Other White	3.4	343
Black, Asian, and Minority Ethnic (BAME)	4.5	577
Unknown	0.5	105
Highest qualification		
No qualification	15.7	1,099
GCSE or lower	26.0	1,678
A level	9.5	712
Degree	37.2	3,324
Unknown	11.7	1,350
Live with a partner		
No	31.4	2,114
Yes	68.6	6,049
Children in the house		
At least one child aged 0-4	6.5	552
At least one school children aged 5-18	18.1	1,488
No school children	75.4	6,123
Key worker		
No	30.8	2,479
Yes	25.2	1,997
Not in paid or self-employed work	44.1	3,687
Has had symptoms that could be coronavirus		
No	89.5	7,266
Yes	10.5	897
Feel lonely		
Hardly ever	65.0	5,488
Sometime	27.4	2,169
Often	7.6	506
Subjective current financial situation		
Living comfortably	35.9	3,261
Doing alright	42.5	3,441
Just about getting by	16.7	1,122
Finding it quite difficult/very difficult	4.9	339
Subjective future financial situation		
Better off	6.1	523
Worse off	15.6	1,245
About the same	78.3	6,395
Prior problem of sleep		
No	84.9	7031
Yes	15.8	1,132
Region		
North East	4.8	291
North West	10.1	774
Yorkshire and The Humber	9.1	712
East Midlands	8.2	659
West Midlands	8.6	669
East of England	10.1	805
London	9.1	658
South East	15.3	1,208
South West	11.0	880
Wales	4.2	454
Scotland	7.3	728
Northern Ireland	2.1	325

Source: authors' analysis, Understanding Society: COVID-19 Study, 2020.
Note: All proportions are weighted using sample weights (wave1 Cross-sectional individual interview weight, beta version). Number of respondents are unweighted.

For peer review only

Table 2. Prevalence of reported sleep loss at each wave of the Understanding Society COVID-19 Study (N=8,163).

		Not at all	No more than usual	Rather more than usual	Much more than usual
Wave 1 (April 2020)	All participants	35.3	42.4	18.3	4.0
	By gender	P<.001			
	Men	45.3	41.0	11.8	1.9
	Women	26.7	43.6	23.9	5.8
	By ethnicity	P<.001			
	British/English/Scottish/Welsh/Northern Irish (White)	35.5	42.3	18.5	3.7
	Other White	34.2	42.6	16.8	6.4
	Black, Asian, and Minority Ethnic (BAME)	18.5	16.8	16.7	6.3
Wave 2 (May 2020)	All participants	33.3	47.5	15.9	3.3
	By gender	P<.001			
	Men	42.6	43.4	11.8	2.2
	Women	24.9	51.2	19.7	4.2
	By ethnicity	P<.001			
	British/English/Scottish/Welsh/Northern Irish (White)	33.8	47.6	15.6	3.0
	Other White	30.2	40.6	23.8	5.4
	Black, Asian, and Minority Ethnic (BAME)	27.8	50.6	15.3	6.3
Wave 3 (June 2020)	All participants	30.1	49.6	16.5	3.8
	By gender	P<.001			
	Men	36.9	47.0	13.1	3.0
	Women	24.1	51.9	19.6	4.4
	By ethnicity	P<.001			
	British/English/Scottish/Welsh/Northern Irish (White)	30.7	49.4	16.4	3.5
	Other White	27.3	44.4	21.2	7.1
	Black, Asian, and Minority Ethnic (BAME)	24.3	55.2	13.9	6.5
Wave 4 (July 2020)	All participants	35.4	48.5	13.6	2.5
	By gender	P<.001			
	Men	44.9	43.6	10.2	1.3
	Women	26.9	52.8	16.6	3.7
	By ethnicity	P=.031			
	British/English/Scottish/Welsh/Northern Irish (White)	35.8	48.4	13.3	2.5
	Other White	30.1	53.1	15.3	1.5
	Black, Asian, and Minority Ethnic (BAME)	34.0	46.0	15.9	4.2
Wave 5 (September 2020)	All participants	31.0	52.3	13.8	2.9
	By gender	P<.001			
	Men	38.4	50.4	9.6	1.6
	Women	24.5	54.0	17.4	4.0
	By ethnicity	P=.004			
	British/English/Scottish/Welsh/Northern Irish (White)	31.3	52.1	13.7	2.9
	Other White	28.8	49.3	19.0	2.9
	Black, Asian, and Minority Ethnic (BAME)	31.5	53.6	12.5	2.3
Wave 6 (November 2020)	All participants	26.2	53.3	16.9	3.7
	By gender	P<.001			
	Men	33.6	51.4	12.8	2.2
	Women	19.7	54.9	20.5	5.0
	By ethnicity	P=.03			
	British/English/Scottish/Welsh/Northern Irish (White)	26.5	53.2	16.7	3.5
	Other White	22.5	53.2	20.6	3.7
	Black, Asian, and Minority Ethnic (BAME)	23.8	52.1	18.9	5.2
Wave 7 (January 2021)	All participants	27.5	53.0	15.9	3.7
	By gender	P<.001			
	Men	34.6	51.7	10.7	3.0
	Women	21.1	54.1	20.6	4.3
	By ethnicity	P<.001			
	British/English/Scottish/Welsh/Northern Irish (White)	28.2	52.3	16.1	3.5
	Other White	23.6	50.5	21.6	4.8
	Black, Asian, and Minority Ethnic (BAME)	20.1	64.2	11.0	4.8

Source: authors' analysis, Understanding Society: COVID-19 Study, 2020.
Note: All proportions are weighted using each wave cross-sectional individual web survey weight, beta version. Number of respondents are unweighted. Pearson Chi-Square test applied.

For peer review only - http://bmjopen.bmj.com/site/about/guidelines.xhtml

Table 3. Self-reported sleep loss and its association with more detailed information on trouble sleeping, July 2020.

	No sleep loss problem	Sleep loss problem	P value
Had trouble sleeping because cannot get to sleep within 30 minutes			P<0.001
Not during the past month	38.3	8.0	
Less than once a week	27.7	10.9	
Once or twice a week	20.6	25.3	
Three or more times a week	7.5	29.0	
More than once most nights	5.8	24.9	
Had trouble sleeping because wake up in the middle of the night or early in the morning			P<0.001
Not during the past month	28.5	2.2	
Less than once a week	23.9	8.8	
Once or twice a week	22.9	28.6	
Three or more times a week	13.4	31.4	
More than once most nights	10.8	28.8	
Had trouble sleeping because cough or snore loudly			P<0.001
Not during the past month	83.9	71.4	
Less than once a week	8.7	10.5	
Once or twice a week	4.2	9.5	
Three or more times a week	1.5	4.1	
More than once most nights	1.3	2.6	
Taken medicine to help sleep			P<0.001
Not during the past month	93.3	78.0	
Less than once a week	2.0	5.1	
Once or twice a week	1.8	5.1	
Three or more times a week	2.7	9.6	
Had trouble staying awake while driving, eating meals, or engaging in social activity			P<0.001
Not during the past month	89.7	71.8	
Less than once a week	6.0	13.3	
Once or twice a week	3.0	10.3	
Three or more times a week	1.1	2.5	
Overall quality of sleep			P<.001
Very good	20.1	0.6	
Fairly good	62.1	31.2	
Fairly bad	15.9	52.9	
Very bad	1.9	15.2	
Average sleep hours	7.09 (SD=1.22)	6.35 (SD=1.20)	P<.001
Number of respondents	6974	1189	

Source: authors' analysis, Understanding Society: COVID-19 Study, 2020. Wave4
Note: All proportions are weighted using wave4 cross-sectional individual web survey weight, beta version. Number of respondents are unweighted. Average sleep hours difference test used ANOVA F test, others used Pearson Chi-Square test.

For peer review only

Table 4. Multivariate modelling of the correlates of sleep loss during the pandemic (odds ratios and 95% CI)

	Model A All respondents				Model B Men		Model C Women	
	A1	A2	A3	A4	B1	B2	C1	C2
Gender								
Men (ref)								
Women	3.2*** (2.8-3.6)		2.1*** (1.9-2.4)	2.8*** (2.4-3.3)				
Ethnicity								
British/English/Scottish/Welsh/Northern Irish (White) (ref)								
Other White		1.5** (1.2-2.1)	1.1 (0.9-1.4)	0.8 (0.5-1.1)	1.3 (0.6-1.9)	0.8 (0.5-1.2)	1.5* (1.1-2.1)	1.3ǂ (0.9-1.7)
Black, Asian, and Minority Ethnic (BAME)		1.4** (1.6-2.4)	0.7*** (0.5-0.8)	0.7* (0.5-0.9)	2.1*** (1.4-3.0)	0.8 (0.6-1.1)	1.1 (0.9-1.5)	0.6*** (0.5-0.8)
Age group								
16-24 (ref)								
25-44			1.2 (0.9-1.6)	1.2 (0.9-1.6)		1.1 (0.6-2.1)		1.2 (0.9-1.7)
45-64			1.3ǂ (0.9-1.7)	1.3ǂ (0.9-1.7)		1.2 (0.7-2.2)		1.3 (0.9-1.8)
65-74			0.9 (0.7-1.2)	0.9 (0.7-1.2)		1.0 (0.5-1.9)		0.8 (0.6-1.2)
75+			0.7* (0.5-0.9)	0.7* (0.5-0.9)		0.6ǂ (0.3-1.1)		0.8 (0.5-1.2)
Highest qualification								
No qualification (ref)								
GCSE or lower			1.1 (0.9-1.3)	1.1 (0.9-1.3)		1.01 (0.8-1.5)		1.2 (0.9-1.4)
A level			1.3** (1.1-1.7)	1.3** (1.1-1.7)		1.5ǂ (0.9-2.1)		1.3 (0.9-1.7)
Degree			1.4*** (1.2-1.7)	1.4*** (1.2-1.7)		1.5* (1.1-1.9)		1.4** (1.2-1.7)
Subjective current financial situation								
Living comfortably (ref)								
Doing alright			1.3*** (1.2-1.4)	1.3*** (1.2-1.4)		1.5*** (1.3-1.8)		1.2** (1.1-1.3)
Just about getting by			2.0*** (1.8-2.3)	2.0*** (1.8-2.3)		2.4*** (1.9-3.0)		1.9*** (1.6-2.2)
Finding it quite difficult/very difficult			3.8*** (3.1-4.6)	3.8*** (3.1-4.6)		4.3*** (3.0-6.1)		3.6*** (2.8-4.5)
Subjective future financial situation								
Better off (ref)								
Worse off			1.5*** (1.3-1.8)	1.5*** (1.3-1.8)		1.6*** (1.2-2.1)		1.5*** (1.2-1.7)
About the same			0.9 (0.8-1.0)	0.9 (0.8-1.0)		0.9 (0.8-1.2)		0.9 (0.8-1.0)
Live with a partner								
No (ref)								
Yes			1.2*** (1.1-1.4)	1.2*** (1.1-1.4)		1.3* (1.0-1.6)		1.2** (1.0-1.3)
Children in the house								
None (ref)								
At least one child aged 0-4			1.1 (0.9-1.3)	1.1 (0.9-1.3)		1.1 (0.8-1.6)		1.1 (0.9-1.4)
At least one school children aged 5-18			1.2** (1.0-1.3)	1.2** (1.0-1.3)		1.2 (0.9-1.5)		1.1ǂ (1.0-1.3)
Key worker								
No (ref)								
Yes			1.0 (0.9-1.1)	1.0 (0.9-1.1)		0.9 (0.8-1.1)		1.1 (0.9-1.2)
Not in paid or self-employed work			0.8** (0.7-0.9)	0.8** (0.7-0.9)		0.7** (0.6-1.0)		0.9 (0.8-1.0)
Has had symptoms that could be coronavirus								
No (ref)								
Yes			1.2** (1.1-1.4)	1.2** (1.1-1.4)		1.2 (0.9-1.5)		1.2* (1.0-1.4)
Feel lonely								
Hardly ever (ref)								
Sometime			3.4*** (3.2-3.7)	3.4*** (3.2-3.7)		4.3*** (3.7-5.0)		3.1*** (2.8-3.4)
Often			12.8*** (11.1-14.7)	12.8*** (11.1-14.7)		15.5*** (11.9-20.2)		11.7*** (9.9-13.8)
Prior problem of sleep								
No (ref)								
Yes			4.5*** (3.9-5.1)	4.5*** (3.9-5.1)		5.1*** (3.9-6.5)		4.1*** (3.5-4.8)
Region								
North East (ref)								
North West			1.2 (0.9-1.6)	1.2 (0.9-1.6)		1.0 (0.6-1.8)		1.3 (0.9-1.9)
Yorkshire and The Humber			1.3ǂ	1.3ǂ		1.1		1.5ǂ

For peer review only - http://bmjopen.bmj.com/site/about/guidelines.xhtml

			**(0.9-1.9)**	**(0.9-1.9)**		(0.6-2.0)		**(1.0-2.2)**
East Midlands			1.2 (0.9-1.7)	1.2 (0.9-1.7)		1.2 (0.7-2.1)		1.3 (0.9-1.9)
West Midlands			**1.4* (1.0-1.9)**	**1.4* (1.0-1.9)**		1.5 (0.8-2.6)		1.4 (0.9-2.0)
East of England			**1.3ǂ (0.9-1.8)**	**1.3ǂ (0.9-1.8)**		1.5 (0.9-2.7)		1.2 (0.8-1.8)
London			**1.7** (1.2-2.4)**	**1.7** (1.2-2.4)**		1.6 (0.9-2.9)		**1.7** (1.2-2.6)**
South East			1.2 (0.9-1.7)	1.2 (0.9-1.7)		1.3 (0.8-2.3)		1.2 (0.8-1.7)
South West			1.1 (0.8-1.6)	1.1 (0.8-1.6)		1.2 (0.7-2.1)		1.1 (0.8-1.6)
Wales			1.2 (0.8-1.6)	1.2 (0.8-1.6)		1.3 (0.7-2.3)		1.1 (0.7-1.7)
Scotland			1.2 (0.9-1.7)	1.2 (0.9-1.7)		1.2 (0.7-2.1)		1.2 (0.8-1.8)
Northern Ireland			1.4 (0.9-1.9)	1.4 (0.9-1.9)		1.4 (0.7-2.6)		1.3 (0.9-2.1)
Month								
April (ref)								
May			**0.7*** (0.6-0.8)**	**0.9 (0.8-1.1)**		0.9 (0.8-1.1)		**0.6*** (0.6-0.7)**
June			**0.7*** (0.7-0.8)**	**1.0 (0.8-1.2)**		1.0 (0.8-1.2)		**0.7*** (0.6-0.7)**
July			**0.5*** (0.4-0.5)**	**0.6*** (0.5-0.7)**		**0.6*** (0.5-0.7)**		**0.4*** (0.4-0.5)**
September			**0.6*** (0.5-0.7)**	**0.7*** (0.6-0.8)**		**0.7*** (0.5-0.8)**		**0.6*** (0.5-0.6)**
November			**0.7*** (0.6-0.8)**	**0.8ǂ (0.7-1.0)**		**0.8ǂ (0.7-1.0)**		**0.7*** (0.6-0.8)**
January			**0.6*** (0.6-0.7)**	**0.8* (0.7-0.9)**		**0.8** (0.6-0.9)**		**0.6*** (0.5-0.7)**
Interaction gender#month								
Women#May				**0.7*** (0.5-0.8)**				
Women#June				**0.6*** (0.5-0.8)**				
Women#July				**0.7** (0.6-0.9)**				
Women#September				0.8 (0.7-1.1)				
Women#November				**0.8* (0.6-0.9)**				
Women#January				**0.7** (0.6-0.9)**				
Interaction ethnicity#month								
BAME#May				**1.4ǂ (1.0-2.1)**				
BAME#June				1.2 (0.8-1.7)				
BAME#July				1.1 (0.7-1.6)				
BAME#September				**0.7ǂ (0.5-1.0)**				
BAME#November				1.0 (0.7-1.5)				
BAME#January				0.9 (0.6-3.4)				
Other White#May				**1.6* (1.0-2.7)**				
Other White#June				1.4 (0.9-2.2)				
Other White#July				1.2 (0.7-2.0)				
Other White#September				1.4 (0.9-2.3)				
Other White#November				1.5 (0.9-2.4)				
Other White#January				1.2 (0.7-1.9)				
LR test vs. logistic model (P value)	*<0.001*	*<0.001*	*<0.001*	*<0.001*	*<0.001*	*<0.001*	*<0.001*	*<0.001*
Number of person-month	*57,141*	*57,141*	*57,141*	*57,141*	*23,926*	*23,926*	*33,215*	*33,215*

Source: authors' analysis, Understanding Society: COVID-19 Study, 2020.
***p<0.001, **p<0.01, *p<0.05, ǂp<0.1

For peer review only

References

1.BBC News. Coronavirus: How the UK is sleeping under lockdown. [cited 2020 June 15]. Available: https://www.bbc.co.uk/news/health-52911395

2.Schwartz JRL, Roth T. Neurophysiology of sleep and wakefulness: basic science and clinical implications. Current Neuropharmacology 2008; **6**: 367–378.

3.Bertisch SM, Pollock BD, Mittleman MA, et al. Insomnia with objective short sleep duration and risk of incident cardiovascular disease and all-cause mortality: Sleep Heart Health Study. Sleep 2018; **41**: zsy047.

4.Liu TZ, Xu C, Rota M, et al. Sleep duration and risk of all-cause mortality: a flexible, non-linear, meta-regression of 40 prospective cohort studies. Sleep Medicine Reviews 2017; **32**: 28–36.

5.Taheri S. The link between short sleep duration and obesity: we should recommend more sleep to prevent obesity. Archives of Disease in Childhood 2006; **91**: 881–884.

6.Kline CE. The bidirectional relationship between exercise and sleep: implications for exercise adherence and sleep improvement. American Journal of Lifestyle Medicine 2014; **8**: 375–379.

7.Hafner M, Stepanek M, Taylor J, Troxel WM, van Stolk C. Why sleep matters—the economic costs of insufficient sleep: a cross-country comparative analysis. Rand Health Quarterly 2017; **6**: 11.

8.Perez-Pozuelo I, Zhai B, Palotti J, et al. The future of sleep health: a data-driven revolution in sleep science and medicine. npj Digital Medicine 2020; **3**: 42.

9.Nugent CN, Black LI. Sleep duration, quality of sleep, and use of sleep medication, by sex and family type, 2013–2014. NCHS Data Brief 2016; **230:** 1–8.

10.Vézina-Im L, Moreno JP, Thompson D, Nicklas TA, Baranowski T. Individual, social and environmental determinants of sleep among women: protocol for a systematic review and meta-analysis. BMJ Open 2017; **7:** e016592.

11.Grandner MA, Williams NJ, Knutson KL, Roberts D, Jean-Louis G. Sleep disparity, race/ethnicity, and socioeconomic position. Sleep Medicine 2016; **18:** 7–18.

12.Jackson CL, Redline S, Emmons KM. Sleep as a potential fundamental contributor to disparities in cardiovascular health. Annual Review of Public Health 2015; **36:** 417–40.

13.Hale L, Rivero-Fuentes E. Negative acculturation in sleep duration among Mexican immigrants and Mexican Americans. Journal of Immigrant Minor Health 2011; **13:** 402–7.

14.Public Health England. Disparities in the risk and outcomes of COVID-19. PHE publications, gateway number: GW-1311. [cited 2020 June 15]. Available: https://assets.publishing.service.gov.uk/government/uploads/system/uploads/attachment_data/file/892085/disparities_review.pdf

15.Evandrou M, Falkingham J, Feng Z, Vlachantoni A. Ethnic inequalities in limiting health and self-reported health in later life revisited. Journal of Epidemiology and Community Health 2016; **70:** 653-662.

For peer review only - http://bmjopen.bmj.com/site/about/guidelines.xhtml

16.Joyce R, Xu X. Shutdowns during the coronavirus crisis: which workers are most exposed? IFS Briefing Note BN279. [cited 2020 July 10]. Available: https://www.ifs.org.uk/uploads/BN278-Sector-Shutdowns.pdf

17.Andrew A, Cattan S, Dias MC, et al. How are mothers and fathers balancing work and family under lockdown? IFS Briefing Note BN290. [cited 2020 July 10]. Available: https://www.ifs.org.uk/uploads/BN290-Mothers-and-fathers-balancing-work-and-life-under-lockdown.pdf

18.BBC News 6th April 2020 'Coronavirus: Domestic abuse calls up 25% since lockdown, charity says' [cited 2020 July 10]. Available: https://www.bbc.co.uk/news/uk-52157620

19.University of Essex, Institute for Social and Economic Research. (2021). Understanding Society: COVID-19 Study, 2020-2021. [data collection]. 8th Edition. UK Data Service. SN: 8644, 10.5255/UKDA-SN-8644-8.

20.University of Essex, Institute for Social and Economic Research. Understanding Society: Waves 1-9, 2009-2018 and Harmonised BHPS: Waves 1-18, 1991-2009. [data collection]. 12th Edition. UK Data Service. 2020; SN: 6614, Available: http://doi.org/10.5255/UKDA-SN-6614-13

21.Grandner MA, Jackson NJ, Izci-Balserak B, et al. Social and behavioral determinants of perceived insufficient sleep. Frontiers in Neurology 2015; **6**: 112.

22.Department of Health and Social Care. Guidance Coronavirus (COVID-19): getting tested. [cited 2020 June 15]. Available: https://www.gov.uk/guidance/coronavirus-covid-19-getting-tested.

23.Bell1 A, Fairbrother M, Jones K. Fixed and random effects models: making an informed choice. Quality & Quantity 2018 https://doi.org/10.1007/s11135-018-0802-x.

24.StataCorp. Stata Statistical Software: Release 15. 2017.College Station, TX: StataCorp LLC.

25.Li SH, Graham BM. Why are women so vulnerable to anxiety, trauma-related and stress-related disorders? The potential role of sex hormones. Lancet Psychiatry 2017; **4:** 73–82.

26.Office for National Statistics (ONS). Coronavirus and anxiety, Great Britain: 3 April 2020 to 10 May 2020. [cited 2020 June 15]. Available: https://www.ons.gov.uk/peoplepopulationandcommunity/wellbeing/articles/coronavirusandanxietygreatbritain/3april2020to10may2020

27.Office for National Statistics (ONS). Coronavirus and key workers in the UK. [cited 2020 June 15]. Available: https://www.ons.gov.uk/employmentandlabourmarket/peopleinwork/earningsandworkinghours/articles/coronavirusandkeyworkersintheuk/2020-05-15

28.Gomez-leon M, Evandrou M, Falkingham J, Vlachantoni A. The dynamics of social care and employment in mid-life. Ageing & Society 2019; **39**: 381-408.

For peer review only - http://bmjopen.bmj.com/site/about/guidelines.xhtml

29.Lauderdale DS, Knutson KL, Yan LL, Liu K, Rathouz PJ. Sleep duration: how well do self-reports reflect objective measures? The CARDIA Sleep Study. Epidemiology 2008; **19:** 838–845. doi: 10.1097/EDE.0b013e318187a7b0

For peer review only

For peer review only - http://bmjopen.bmj.com/site/about/guidelines.xhtml

Figure 1. Prevalence of reported sleep loss pre and during the pandemic among all participants aged 16 and above in Understanding Society mainstage survey in 2019 and Understanding Society: COVID-19 Study Wave 1 to Wave 7.

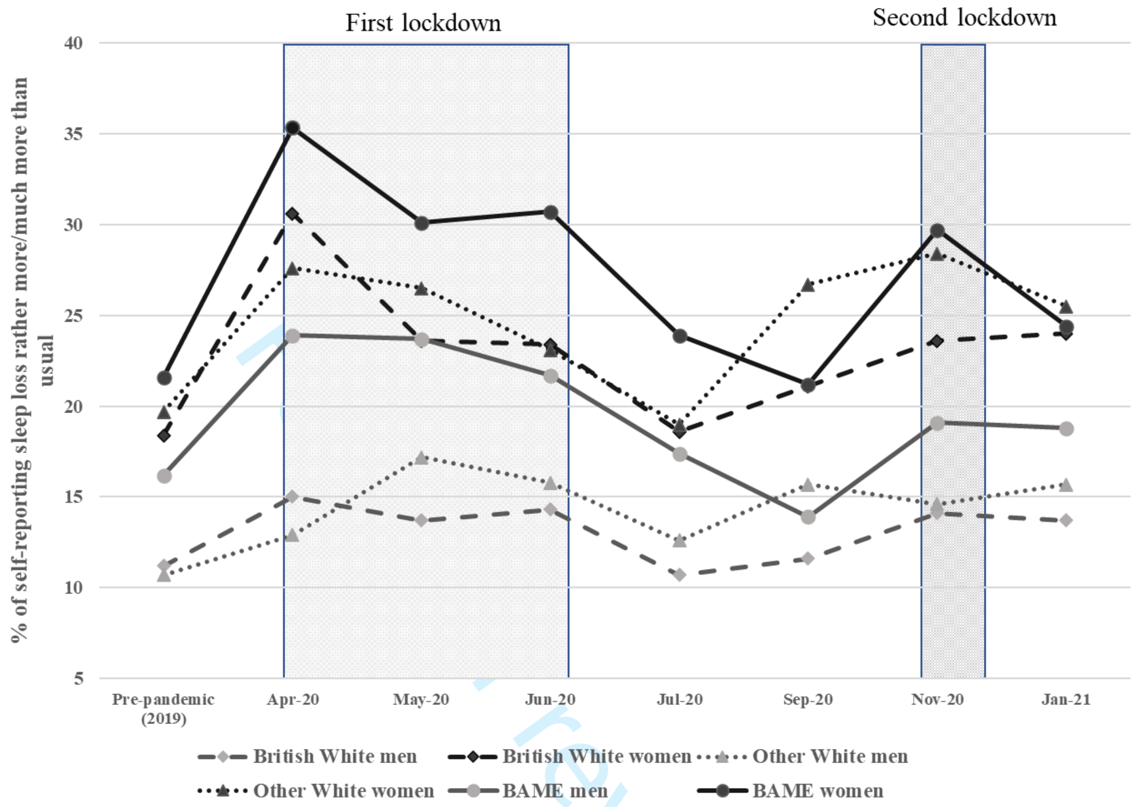

Source: authors' analysis, Understanding Society: COVID-19 Study, 2020.
Note: All proportions are unweighted. Black lines represent women in different ethnicities and grey lines represent men.
Number of respondents: N=29,685 (Pre-pandemic 2019), N=15,668 (2020 April), N=14,154 (2020 May), N=13,437 (2020 June), N=13,075 (2020 July), N= 12,170 (2020 September), N=11,472 (2020 November), N=11,299 (2021 January).

STROBE 2007 (v4) Statement—Checklist of items that should be included in reports of *cohort studies*

**Section/Topic**	**Item #**	**Recommendation**	**Reported on page #**
**Title and abstract**	1	(*a*) Indicate the study's design with a commonly used term in the title or the abstract	1,2
		(*b*) Provide in the abstract an informative and balanced summary of what was done and what was found	2
**Introduction**			
Background/rationale	2	Explain the scientific background and rationale for the investigation being reported	4-5
Objectives	3	State specific objectives, including any prespecified hypotheses	5
**Methods**			
Study design	4	Present key elements of study design early in the paper	5
Setting	5	Describe the setting, locations, and relevant dates, including periods of recruitment, exposure, follow-up, and data collection	5-7
Participants	6	(*a*) Give the eligibility criteria, and the sources and methods of selection of participants. Describe methods of follow-up	6
		(*b*) For matched studies, give matching criteria and number of exposed and unexposed	-
Variables	7	Clearly define all outcomes, exposures, predictors, potential confounders, and effect modifiers. Give diagnostic criteria, if applicable	6-7
Data sources/ measurement	8*	For each variable of interest, give sources of data and details of methods of assessment (measurement). Describe comparability of assessment methods if there is more than one group	
Bias	9	Describe any efforts to address potential sources of bias	6
Study size	10	Explain how the study size was arrived at	6
Quantitative variables	11	Explain how quantitative variables were handled in the analyses. If applicable, describe which groupings were chosen and why	6-7
Statistical methods	12	(*a*) Describe all statistical methods, including those used to control for confounding	7-8
		(*b*) Describe any methods used to examine subgroups and interactions	7-8
		(*c*) Explain how missing data were addressed	7-8
		(*d*) If applicable, explain how loss to follow-up was addressed	6
		(*e*) Describe any sensitivity analyses	8
**Results**			

For peer review only

Participants	13*	(a) Report numbers of individuals at each stage of study—eg numbers potentially eligible, examined for eligibility, confirmed eligible, included in the study, completing follow-up, and analysed	6,8
		(b) Give reasons for non-participation at each stage	6,8
		(c) Consider use of a flow diagram	-
Descriptive data	14*	(a) Give characteristics of study participants (eg demographic, clinical, social) and information on exposures and potential confounders	8-9
		(b) Indicate number of participants with missing data for each variable of interest	6
		(c) Summarise follow-up time (eg, average and total amount)	8
Outcome data	15*	Report numbers of outcome events or summary measures over time	8-9
Main results	16	(*a*) Give unadjusted estimates and, if applicable, confounder-adjusted estimates and their precision (eg, 95% confidence interval). Make clear which confounders were adjusted for and why they were included	9-10
		(*b*) Report category boundaries when continuous variables were categorized	-
		(*c*) If relevant, consider translating estimates of relative risk into absolute risk for a meaningful time period	-
Other analyses	17	Report other analyses done—eg analyses of subgroups and interactions, and sensitivity analyses	10
**Discussion**			
Key results	18	Summarise key results with reference to study objectives	10
**Limitations**			
Interpretation	20	Give a cautious overall interpretation of results considering objectives, limitations, multiplicity of analyses, results from similar studies, and other relevant evidence	10-11
Generalisability	21	Discuss the generalisability (external validity) of the study results	11
**Other information**			
Funding	22	Give the source of funding and the role of the funders for the present study and, if applicable, for the original study on which the present article is based	3

*Give information separately for cases and controls in case-control studies and, if applicable, for exposed and unexposed groups in cohort and cross-sectional studies.

Note: An Explanation and Elaboration article discusses each checklist item and gives methodological background and published examples of transparent reporting. The STROBE checklist is best used in conjunction with this article (freely available on the Web sites of PLoS Medicine at http://www.plosmedicine.org/, Annals of Internal Medicine at http://www.annals.org/, and Epidemiology at http://www.epidem.com/). Information on the STROBE Initiative is available at www.strobe-statement.org.

For peer review only

Appendix Table A. Characteristics of full wave 1 (April 2020) COVID-19 sample and analytical (follow-up) sample

	**Full wave 1 sample**	**Analytical sample**	**P***
**Age, mean (SD)**	50.1 (18.2) (17,452)	54.9 (16.2) (8163)	<.001
**Age, % (n)**			<.001
16-24	10.2 (1,543)	4.6 (309)	-
25-44	28.6 (4,734)	21.6 (1587)	-
45-64	36.9 (7,028)	41.7 (3571)	-
65-74	15.0 (2,925)	22.4 (1972)	-
75+	9.4 (1,222)	9.7 (724)	-
**Gender, % (n)**			<.001
**Men**	48.0 (7,287)	46.2 (3418)	-
**Women**	52.0 (10,165)	53.8 (4745)	-
**Ethnicity, % (n)**			<.001
British/English/Scottish/Welsh/Northern Irish (White)	86.5 (14,029)	91.4 (7138)	-
Other White	3.7 (779)	3.3 (343)	-
Black, Asian, and Minority Ethnic (BAME)	8.7 (2,044)	4.8 (577)	-
**Subjective curent Financial situation, % (n)**			<.001
Living comfortably	29.6 (5,815)	36.0 (3261)	
Doing alright	40.1 (7,045)	42.5 (3441)	
Just about getting by	17.2 (2,585)	16.6 (1188)	
Finding it quite difficult/very difficult	6.2 (891)	4.9 (339)	
**Sleep loss, % (n)**			<.001
No	75.2 (12,008)	77.8 (6371)	-
Yes	24.8 (3,919)	22.2 (1792)	-

Source: authors' analysis, Understanding Society: COVID-19 Study, 2020.
* p-value for comparison between participants in the initial sample who did and did not respond to follow-up.
Note: All proportions are weighted using sample weights. Number of respondents are unweighted. Mean age difference test used ANOVA F test, others used Pearson Chi-Square test.

For peer review only

Appendix Table B. Sleep loss comparison before and after the COVID-19 pandemic by sex and ethnicity (N=8,163).

	Sleep loss before the COVID-19 pandemic (2019)	Sleep loss after the COVID-19 pandemic (April 2020)
Total	13.9	22.0
Sex	P<0.001	P<0.001
Men	10.2	13.5
Women	16.5	28.0
Ethnicity	P=0.05	P=0.438
British/English/Scottish/Welsh/Northern Irish (White)	13.5	21.7
Other White	15.5	23.3
Black, Asian, and Minority Ethnic (BAME)	17.2	24.3

Source: authors' analysis, Wave 1 of Understanding Society: COVID-19 Study, 2020.

Note: All proportions and number of respondents are unweighted. Pearson Chi-Square test applied.

For peer review only

Appendix Table C. Characteristics of participants, by ethnicity (N=8,163).

	British/English/Scottish/Welsh/Northern Irish (White)	Other White	Black, Asian, and Minority Ethnic (BAME)
Age group **% (n)**	P<0.001		
16-24	4.6 (199)	1.0 (1)	12.2 (54)
25-44	21.1 (1,286)	26.1 (76)	30.4 (194)
45-64	41.5 (3,114)	50.2 (174)	43.7 (268)
65-74	22.7 (1,850)	16.7 (72)	10.4 (48)
75+	10.1 (689)	5.9 (20)	3.3 (13)
Gender **% (n)**	P=0.111		
Men	46.2 (3,009)	38.6 (122)	47.0 (244)
Women	53.8 (4,129)	61.4 (221)	53.0 (333)
Subjective current financial situation **% (n)**	P<0.001		
Living comfortably	36.8 (2,949)	33.7 (127)	21.9 (149)
Doing alright	42.5 (3,016)	42.1 (147)	40.4 (228)
Just about getting by	16.1 (915)	19.3 (54)	28.1 (140)
Finding it quite difficult/very difficult	4.6 (258)	5.0 (15)	9.6 (60)
Subjective future financial situation **% (n)**	P<0.001		
About the same	79.3 (5,640)	66.8 (249)	68.9 (420)
Better off	5.8 (450)	10.4 (28)	6.7 (37)
Worse off	14.9 (1,048)	22.8 (66)	24.4 (120)
Children in the house **% (n)**	P<0.001		
No school aged child	76.3 (5,503)	68.5 (239)	60.0 (314)
At least one child aged 0-4	6.3 (449)	5.4 (24)	11.5 (72)
At least one school children aged 5-18	17.3 (1,186)	26.1 (80)	28.5 (191)
Key worker **% (n)**	P<0.001		
No	30.4 (2,130)	35.1 (121)	29.6 (189)
Yes	24.9 (1,709)	30.2 (87)	28.1 (177)
Not in paid or self-employed work	44.7 (3,299)	34.7 (135)	42.2 (211)
Has had symptoms that could be coronavirus **% (n)**	P=0.012		
No	89.8 (6,375)	90.6 (308)	83.7 (489)
Yes	10.2 (763)	9.4 (35)	16.3 (88)
Feel lonely **% (n)**	P<0.001		
Hardly ever	65.5 (4,889)	64.4 (228)	58.3 (329)
Sometime	27.0 (1,821)	26.2 (94)	33.2 (203)
Often	7.4 (428)	9.4 (21)	8.5 (45)
Prior sleep loss **% (n)**	P=0.557		
No	84.9 (6,176)	82.7 (290)	87.0 (478)
Yes	15.1 (962)	17.3 (53)	13.0 (99)
Region **% (n)**	P<0.001		
North East (ref)	5.0 (278)	1.5 (4)	0.7 (4)
North West	10.3 (684)	5.9 (20)	8.9 (62)
Yorkshire and The Humber	9.4 (633)	5.4 (19)	7.8 (54)
East Midlands	8.5 (611)	4.5 (14)	4.4 (30)
West Midlands	8.8 (563)	3.5 (21)	10.0 (76)
East of England	10.5 (725)	9.9 (28)	4.4 (41)
London	7.1 (373)	21.8 (65)	40.4 (210)
South East	15.7 (1,094)	12.4 (46)	11.5 (58)
South West	11.4 (820)	9.4 (30)	6.7 (20)
Wales	4.4 (423)	5.0 (16)	1.9 (12)
Scotland	7.5 (693)	5.4 (15)	3.3 (8)
Northern Ireland	1.6 (241)	15.3 (65)	0.1 (2)

Source: authors' analysis, Understanding Society: COVID-19 Study, 2020.
Note: All proportions are weighted using sample weights. The percentages are 100% within each ethnic group. Number of respondents are unweighted. Pearson Chi-Square test applied.

For peer review only

Appendix Table D. Sensitivity analyses: odds ratios and 95% CI of mixed-effects ordered logistic regressions.

	Model A All respondents			
	A1	A2	A3	A4
Gender				
Men (ref)				
Women	**3.6*** **		**2.5*** **	**3.4*** **
	**(3.2-4.0)**		**(2.2-2.7)**	**(3.0-3.9)**
Ethnicity				
British/English/Scottish/Welsh/Northern Irish (White) (ref)				
Other White		**1.6** **	1.1	0.9
		**(1.2-2.1)**	(0.9-1.4)	(0.7-1.2)
Black, Asian, and Minority Ethnic (BAME)		**1.6*** **	0.9	**0.8* **
		**(1.3-2.1)**	(0.7-1.1)	**(0.6-0.9)**
Month				
April (ref)				
May			0.9	**1.1* **
			(0.9-1.0)	**(1.0-1.3)**
June			**1.1** **	**1.4*** **
			**(1.0-1.2)**	**(1.3-1.6)**
July			**0.7*** **	**0.8*** **
			**(0.7-0.8)**	**(0.5-0.6)**
September			**0.9**	**1.2** **
			**(0.9-1.0)**	**(1.1-1.4)**
November			**1.2*** **	**1.5*** **
			**(1.1-1.3)**	**(1.3-1.6)**
January			1.1	**1.3*** **
			(0.9-1.1)	**(1.2-1.5)**
Interaction gender#month				
Women#May				**0.7*** **
				**(0.6-0.8)**
Women#June				**0.6*** **
				**(0.5-0.7)**
Women#July				**0.7*** **
				**(0.6-0.8)**
Women#September				**0.7*** **
				**(0.6-0.8)**
Women#November				**0.7*** **
				**(0.6-0.8)**
Women#January				**0.7*** **
				**(0.6-0.8)**
Interaction ethnicity#month				
BAME#May				**1.3* **
				**(1.0-1.7)**
BAME#June				1.2
				(0.9-1.5)
BAME#July				**1.3* **
				**(1.0-1.7)**
BAME#September				0.9
				(0.7-1.2)
BAME#November				1.1
				(0.8-1.4)
BAME#January				1.2
				(0.9-1.6)
Other White#May				1.3
				(0.9-1.8)
Other White#June				1.2
				(0.9-1.7)
Other White#July				1.0
				(0.7-1.5)
Other White#September				1.2
				(0.9-1.8)
Other White#November				1.2
				(0.9-1.8)
Other White#January				1.1
				(0.8-1.6)
LR test vs. ologit model (P value)	*<0.001*	*<0.001*	*<0.001*	*<0.001*
Number of person-month	*57,141*	*57,141*	*57,141*	*57,141*

Source: authors' analysis, Understanding Society: COVID-19 Study, 2020.
***p<0.001, **p<0.01, *p<0.05
Notes: Model A1 and A2 were bivariate models with gender or ethnicity in the model. Model A3 and A4 were multivariate models, controlling for age, highest qualification, subjective current financial situation, subjective future financial situation, live with a partner, children in the house, key worker, felt lonely, prior problem of sleep, region and month.

For peer review only

A prospective longitudinal study of "Sleepless in Lockdown": unpacking differences in sleep loss during the coronavirus pandemic in the UK (bmjopen-2021-053094)

Dear Editor,

Thank you for the useful comments on our paper. Below we outline how each of the reviewer's comments has been addressed in the revised paper.

In addition, thanks to the newly released survey data, we were able to cover in our analysis an extended period during the pandemic, using data from seven waves (compared to four waves included in the previous manuscript) of the Understanding Society (USoc) COVID-19 Study collected from April 2020 to January 2021 and linked to 2019 pre-pandemic data from the mainstage interviews. We have update all the tables to reflect this. Figure 1 has been added to describe the overall trend across the lockdowns, and Table 3 has been added to examine the validation of sleep loss with detailed trouble sleeping (information only available in wave 4). The results and conclusions have also been adjusted, but show similar patterns to our original submission.

Reviewer: 1
Dr. Lydi-Anne Vézina-Im, Baylor College of Medicine Comments to the Author:
The topic of this article is highly relevant and the article was very well-written. I have a few comments that may contribute to improve the manuscript.

Major Comments
1) In the abstract on page 3: Please define sleep loss in the abstract. For example what specific criterion was used to consider a loss of sleep (a loss of 30 minutes or 60 minutes)?

Response: We thank Dr. Lydi-Anne Vézina-Im for the comments and suggestions. In our paper, sleep loss is a perceived self-reported subjective measure. It is hard to set a specific criterion such as a sleep loss of 30 or 60 minutes. Nevertheless, we now have added descriptive analysis (Table 3, Page 15) to check the general sleep loss validation with certain other sleep health variables (e.g. actual sleep hours per night, trouble getting to sleep within 30 minutes, and others). The results indicate that the general sleep loss measurement is a good proxy for this wider set of sleep-related issues. (Page 9)

Since the detailed sleep health information is only available in one wave of the survey data, we could not use the variable for the longitudinal analysis. A more detailed explanation on this issue is included below in response to comment 3).

2) In the study design and population section on page 6: Were pregnant women excluded from the study? Pregnancy, menstrual cycle and menopause are all potential biological explanations why women usually report shorter/poorer sleep quality compared to men. This brings me to another question, did the authors control for where women were in their menstrual cycle and/or if they had reached menopause? These two factors could affect sleep loss among women and explain sex differences on that variable.

Response: In the analysis, we didn't exclude pregnant women. We appreciate that sleep quality may decline (e.g. onset insomnia and frequent awakening) towards the third trimester of one's pregnancy, however the survey data does not provide such detailed information. Also, given the data availability constraint, we were not able to consider a menopause or menstrual cycle variable in our analysis. Instead, age and gender are controlled for in our statistical models. Since the average age of natural menopause is around 50–51, we hope that, to some extent, we could have captured this biological aspect in our analysis.

For peer review only

3) In the procedures on page 7: Before the question asking participants if they had experienced sleep loss over the last few weeks, was a definition of sleep loss given? This is important as if no definition of sleep loss was provided, participants might have used different definitions while answering the question. For example, one person could have considered a loss of 10 minutes of sleep not being enough to be considered a sleep loss, while for another person a loss of > 1 minutes might have been enough to be considered a sleep loss. If no definition of sleep loss was provided in the questionnaire, this should be added as a limitation of the study as there might have been variability in the personal definitions used by participants. It also seems that the measure used to assess sleep loss was not validated. If this is the case, this should also be added as a limitation of the study. I would also specify that it would have been preferable to have a more comprehensive measure of sleep, which measured precisely sleep duration and quality.

Response: We agree with this comment and have added descriptive analysis (Table 3, Page 15) to check the general sleep loss measurement validation with certain other sleep health variables. In addition to the general sleep loss question, in the Wave 4 COVID-19 study, participants were asked a more detailed set of questions about sleep including: How many hours of actual sleep did you usually get per night during the last month? How often have you had trouble sleeping because you cannot get to sleep within 30 minutes? How often have you had trouble sleeping because you wake up in the middle of the night or early in the morning? These more comprehensive measures were compared against the general sleep loss measure as part of the descriptive analyses. (Page 6)

The results show that, on average, those who reported problems with sleep slept around 45 minutes less than those who did not have trouble sleeping (Table 3). The majority of respondents reporting sleep loss had trouble getting to sleep within 30 minutes or reported waking up in the middle of the night or early in the morning. They were also more likely to rate their overall quality of sleep as fairly bad or very bad. They were also more likely to report having trouble sleeping because of having a cough or snoring loudly, taking medicine to help them sleep, and having trouble staying awake while driving, eating meals, or engaging in social activity (Table 3), indicating that the general sleep loss question is a good proxy for these wider set of sleep-related issues. (Page 9)

4) In the multivariate analysis section on line 25 on page 10: I am surprised being a woman of BAME heritage was a protective factor of sleep loss. This seems to contradict the rest of the results where being of BAME heritage is a risk factor for sleep loss. You would think the cumulative effect of these two risk factors for sleep loss (being a woman and of BAME heritage) would make this section of the population be at the most risk of developing sleep loss. Do the authors have any hypothesis to explain this result? If so, please add this hypothesis/potential explanation in the discussion.

Response: Our descriptive analysis does show that the proportion of sleep loss among women of BAME heritage was the highest, compared with that of other ethnic groups. We added Figure 1 (Page 21) to illustrate the level of reported sleep loss by gender and ethnicity among all participants aged 16 and above in the USoc mainstage survey in 2019 and in the USoc: COVID-19 Study Wave 1 to Wave 7. Overall, women reported much higher levels of sleep loss than men; and respondents from ethnic minority groups had a higher prevalence of sleep loss than British White respondents among both men and women, with BAME women experiencing the highest prevalence. (Page 9)

However, this observed pattern is not due to being a woman of BAME heritage per se, but because of such women's associated demographic characteristics and socioeconomic disadvantages. Therefore, once the multivariate statistic models control for such factors (Table 4, Page 16), the association between being a woman of BAME heritage and sleep loss reverses. Biopsychological reasons behind this association are beyond the scope of this study. However, when a BAME woman and a White British woman had a similar demographic profile and socioeconomic status, life stressors in the

For peer review only

context of the COVID19 pandemic, which usually induce negative cognitive-affective dispositions, seem not to influence the sleep process of BAME women as much as they do among White British women. It might be possible that BAME women are better appraisers of daily stressors or displayed/ developed more effective stress coping skills during the pandemic, which would reduce their vulnerability to sleep loss, particularly where anxiety constitutes the dominant feature (Morin et al., 2003).

Reference: Morin, C.M., Rodrigue, S., Ivers, H. Role of stress, arousal, and coping skills in primary insomnia. Psychosom Med. 2003;65:259–267. doi: 10.1097/01.PSY.0000030391.09558.A3.

5) In the discussion on lines 16-19 on page 11: Do the authors have any hypothesis why being a member of BAME community was associated with a reduced chance of sleep loss once all risk factors for sleep loss were controlled for in the analyses? Table C in the appendix reports that slightly more BAME individuals were women (54.4% vs. 53.6 %) and slightly more were key workers (26.4% vs. 25.9%) so you would think BAME individuals would be more at risk for sleep loss since women and key workers were found to be more at risk for sleep loss. Do the authors any other potential explanation for their results? If so, please add this hypothesis/potential explanation in the discussion.

Response: As we described above for comment 4), our descriptive analysis does show that respondents from ethnic minority groups had a higher prevalence of sleep loss than White British respondents among both men and women (Page 9), as illustrated in Figure 1 (Page 21).

However, this observed pattern is not because of being a person of BAME heritage per se, but because of such individuals' associated demographic characteristics and socioeconomic disadvantages (e.g., slightly more BAME individuals were women and slightly more were key workers). As a result, once the multivariate statistic models control for such factors (Table 4, Page 16), being a member of a BAME community was associated with a reduced chance of sleep loss. Separate models among men and women (Table 4, Page 16) show that the reverse association happens among BAME women only. Biopsychological reasons behind this association are beyond the scope of this study. The potential explanation echoes the above response for comment 4). When a BAME woman and a White British woman had a similar demographic profile and socioeconomic status, life stressors in the context of the COVID19 pandemic, which usually induce negative cognitive-affective dispositions, seem not to influence the sleep process of BAME women as much as they do to White British women. It might be possible that BAME women are better appraisers of daily stressors or have displayed/developed more effective stress coping skill during the pandemic, which would reduce their vulnerability to sleep loss, particularly where anxiety constitutes the dominant feature (Morin et al., 2003).

Minor Comments

1) In the abstract, on line 5 on page 3: Please put "Covid-19" all in capitals for consistency with the rest of the abstract.

Response: It is now corrected to show in capitals.

2) In the strengths and limitations section on line 42 on page 3: I am not sure what is meant by "interested prospective associations". Please clarify.

Response: "interested prospective associations" are the associations we examine----- social determinants and self-reported sleep loss over the observation period.

3) In the introduction on line 38 on page 5: I think there is a mistake. It is mentioned that BAME stands for black and minority ethnic while elsewhere in the manuscripts, it stands for Black, Asian

For peer review only

and minority ethnic like in the abstract (line 6 on page 3), in the procedures (line 53 on page 7) and in the tables 1, 2 and 3 (pages 12-15).

Response: It is now corrected as 'Black, Asian and minority ethnic'.

4) In the procedures on line 47 on page 7: There is a mistake. It should be "hypothesize" instead of "hypothesis" (i.e., we hypothesize may be associated…).

Response: Thank you, we think 'hypothesis' here is a noun rather than a verb.

For peer review only

BMJ Open

A prospective longitudinal study of “Sleepless in Lockdown”: unpacking differences in sleep loss during the coronavirus pandemic in the UK

Journal:	*BMJ Open*
Manuscript ID	bmjopen-2021-053094.R1
Article Type:	Original research
Date Submitted by the Author:	15-Nov-2021
Complete List of Authors:	Falkingham, J; ESRC Centre for Population Change, Faculty of Social Sciences, University of Southampton<br>Evandrou , Maria ; University of Southampton Centre for Research on Ageing, Faculty of Social Sciences; ESRC Centre for Population Change, Faculty of Social Sciences, University of Southampton<br>Qin, Min; ESRC Centre for Population Change, Faculty of Social Sciences, University of Southampton<br>Vlachantoni, Athina; University of Southampton Centre for Research on Ageing, Faculty of Social Sciences; ESRC Centre for Population Change, Faculty of Social Sciences, University of Southampton
**Primary Subject Heading**:	Epidemiology
Secondary Subject Heading:	Global health
Keywords:	COVID-19, EPIDEMIOLOGY, MENTAL HEALTH

SCHOLARONE™
Manuscripts

I, the Submitting Author has the right to grant and does grant on behalf of all authors of the Work (as defined in the below author licence), an exclusive licence and/or a non-exclusive licence for contributions from authors who are: i) UK Crown employees; ii) where BMJ has agreed a CC-BY licence shall apply, and/or iii) in accordance with the terms applicable for US Federal Government officers or employees acting as part of their official duties; on a worldwide, perpetual, irrevocable, royalty-free basis to BMJ Publishing Group Ltd ("BMJ") its licensees and where the relevant Journal is co-owned by BMJ to the co-owners of the Journal, to publish the Work in this journal and any other BMJ products and to exploit all rights, as set out in our licence.

The Submitting Author accepts and understands that any supply made under these terms is made by BMJ to the Submitting Author unless you are acting as an employee on behalf of your employer or a postgraduate student of an affiliated institution which is paying any applicable article publishing charge ("APC") for Open Access articles. Where the Submitting Author wishes to make the Work available on an Open Access basis (and intends to pay the relevant APC), the terms of reuse of such Open Access shall be governed by a Creative Commons licence – details of these licences and which Creative Commons licence will apply to this Work are set out in our licence referred to above.

Other than as permitted in any relevant BMJ Author's Self Archiving Policies, I confirm this Work has not been accepted for publication elsewhere, is not being considered for publication elsewhere and does not duplicate material already published. I confirm all authors consent to publication of this Work and authorise the granting of this licence.

For peer review only

A prospective longitudinal study of "Sleepless in Lockdown": unpacking differences in sleep loss during the coronavirus pandemic in the UK

Jane Falkingham, Maria Evandrou, Min Qin and Athina Vlachantoni

Authors' affiliations

Jane Falkingham (Corresponding Author)
ESRC Centre for Population Change, Faculty of Social Sciences, University of Southampton, Southampton, UK
j.c.falkingham@soton.ac.uk
07872423609

Maria Evandrou
Centre for Research on Ageing and ESRC Centre for Population Change, University of Southampton, Southampton, UK

Min Qin
ESRC Centre for Population Change, Faculty of Social Sciences, University of Southampton, Southampton, UK

Athina Vlachantoni
Centre for Research on Ageing and ESRC Centre for Population Change, University of Southampton, Southampton, UK

For peer review only

Abstract

Objectives: COVID-19 is having a disproportionate impact on Black, Asian, and Minority Ethnic (BAME) groups and women. Concern over direct and indirect effects may also impact upon sleep. We explore the levels and social determinants of self-reported sleep loss amongst the UK population during the pandemic, focusing on ethnic and gender disparities.
Setting: This prospective longitudinal study analysed data from seven waves of the Understanding Society (USoc) COVID-19 Study collected from April 2020 to January 2021 linked to pre-pandemic data from the 2019 mainstage interviews, providing baseline information about the respondents prior to the pandemic.
Participants: The analytical sample included 8,163 respondents aged 16 and above who took part in all seven waves with full information on sleep loss, defined as experiencing 'rather more' or 'much more' than usual sleep loss due to worry, providing 57,141 observations.
Primary outcome measures: Self-reported sleep loss. Mixed-effects regression models were fitted to consider within- and between-individual differences.
Results: Women were more likely to report sleep loss than men (odds ratio [OR] 2.1 [95% CI 1.9–2.4]) over the ten-month period. Being female, having young children, perceived financial difficulties and COVID-19 symptoms were all predictive of sleep loss. Once these covariates were controlled for, the bivariate relationship between ethnicity and sleep loss (1.4 [1.6–2.4]) was reversed (0.7 [0.5–0.8]). Moreover, the strength of the association between gender and ethnicity and the risk of sleep loss varied over time, being weaker among women in July (0.6 [0.5–0.7]), September (0.7 [0.6–0.8]), November (0.8 [0.7–1.0]) and January 2021 (0.8 [0.7–0.9]) compared with April 2020, but positively stronger among BAME individuals in May (1.4 [1.0–2.1]), weaker only in September (0.7 [0.5–1.0]).
Conclusions: The pandemic has widened sleep deprivation disparities, with women with young children, COVID-19 infection and BAME individuals experiencing sleep loss, which may adversely affect their mental and physical health.

Strengths and limitations of this study

- This is a large-scale community-based prospective longitudinal study.
- We examined the trends and patterns of sleep problems and the impact of known correlates of being infected with, or affected by, COVID-19 including ethnicity and gender.
- Sleep loss was measured by self-reports. As no definition of sleep loss was provided, participants may have used different definitions while answering the question.
- Attrition could have confounded the interested prospective associations.

For peer review only - http://bmjopen.bmj.com/site/about/guidelines.xhtml

Funding: This research was supported by the Economic and Social Research Council Centre for Population Change (grant number ES/K007394/1) at the University of Southampton.

Keywords: COVID-19; Pandemic; Sleep Loss; Mental Health; Gender; Ethnicity

Competing Interest Statement
The authors have declared no competing interest.

Ethics approval
This study uses secondary data for the collection of which ethical approval has been obtained by the survey team. All relevant ethical guidelines at the University of Southampton have been followed.

Authors' contributions
All authors (JF, ME, MQ, AV) contributed equally to the initial discussion of the idea behind the manuscript, to the design of the manuscript, to the analysis plan and to the redrafting and finalisation of the manuscript. MQ conducted the statistical analysis for the paper and drafted sections of the initial draft of the manuscript.

Role of the funding source
This research was supported by the Economic and Social Research Council Centre for Population Change (grant number ES/K007394/1) at the University of Southampton. This grant paid for the staff time dedicated to the discussion around the manuscript and the writing of the manuscript. None of the authors has been paid to write this article by a pharmaceutical company or other agency. JF who is the corresponding author had full access to all the data in the study and had final responsibility for the decision to submit the paper for publication.

Data availability statement
No additional data available.

For peer review only

A prospective longitudinal study of "Sleepless in Lockdown": unpacking differences in sleep loss during the coronavirus pandemic in the UK

Introduction

The coronavirus disease (COVID-19) is impacting upon physical and mental health globally. Sleep problems associated with increased psychosocial stressors induced by the coronavirus itself, and as a result of the social distancing measures that have been introduced to manage the virus, are emerging as a significant outcome of the COVID-19 crisis. According to a report last summer, more than half of the UK population has struggled with sleep during the first lockdown [1]. Sleep has long been recognised as an essential determinant of human health and performance. Good sleep restores energy, promotes healing, interacts with the immune system and impacts upon behaviour [2]. Even acute sleep deprivation can impair judgement and cognitive performance, while persistent deviations have been linked to disease development and increased mortality [3, 4]. During the pandemic, lack of sleep may itself have had knock-on effects on people's capacity to be resilient. However, to date relatively limited research has been conducted on sleep deprivation during the pandemic.

Sleep is known to be regulated by circadian rhythms, sleep-wake homoeostasis and cognitive-behavioural influences [3]. With regards to environmental, behavioural and health determinants, poor sleep has been associated with stress, anxiety, work pressures, financial concerns, mental and physical impairments, and physical activity [5, 6, 7, 8]. Previous studies have found that women were more likely than men to have trouble falling and staying asleep frequently, or to have insufficient sleep [9, 10]. The relationship between ethnicity and sleep is complicated due to the broader social and environmental factors determining group differences in sleep behaviours and the structural relationships between these factors and ethnicity [11]. Some studies reported that inadequate sleep duration and poorer sleep were more prevalent among low-income and Black, Asian and Minority Ethnic (BAME) communities [12], whereas others have failed to find this association [13].

In understanding the relationship between COVID-19 and sleep, it is helpful to conceptually distinguish between those factors linked to being *infected* with COVID-19 and those associated with the policy responses and measures introduced to manage the pandemic that have *affected* everyday life. Although it is still relatively early in our understanding of COVID-19, research by Public Health England 2020 has highlighted that older people, men and individuals from BAME groups are all at increased risk of developing a severe response to the virus and to die from it [14]. The reasons for the heightened risk amongst certain ethnic groups remains unresolved, but potential contributors include the disproportionate representation of BAME individuals in some high risk occupations including front line health care work, as well as wider environmental factors that, interwoven with issues of inequality, deprivation and structural racism, manifest in longstanding ethnic disparities in health [15]. A priori, we might expect those groups facing the greatest health risks from the virus to report increased sleep loss due to worry and thus to observe differences across ethnic groups.

The public health actions taken to control the spread of the virus have, however, impacted all domains of life and thus affected all individuals. On 23rd March 2020, the UK went into lockdown

For peer review only - http://bmjopen.bmj.com/site/about/guidelines.xhtml

in an unprecedented attempt to limit the spread of coronavirus, with the Government mandating all those who could to work at home, closing schools, restaurants and all but essential shops, and advising the population to stay at home and limit contact with other individuals outside their household. On 5th November, a second national lockdown came into force in England, followed by the third nationwide lockdown on 5th January 2021, the phased end of which is scheduled for 21 June. The resultant move to home working and learning and, for some, the loss of work altogether, along with limited social contact and increased isolation, may all be anticipated to affect mental well-being and the ability to sleep. Preliminary evidence points towards the young and women being disproportionally affected, with women being more likely than men to be working in sectors that were locked down [16] and mothers being more likely to be interrupted whilst working from home than fathers [17]. Lockdown has also resulted in increased instances of domestic violence; the UK domestic abuse organisation, Refuge, reported a 25 per cent increase in calls and online requests since the first lockdown began in March 2020 [18]. Given this, we might anticipate a gender differential in increased sleep loss, with women being disproportionately affected by lockdown compared with men.

This study aims to provide novel evidence regarding patterns of self-reported increased sleep loss due to worry during the first four weeks of the COVID-19 related lockdown in the UK. Using recently collected nationally representative survey data, the research provides the first estimates of the prevalence and incidence of increased sleep loss since the coronavirus pandemic. It attempts to unpack the impact of factors associated with being *infected* and being *affected* by COVID-19, with a particular focus on the extent to which the pandemic has exacerbated differentials in sleep loss by ethnicity and gender.

Methods

Study design and population

We analysed data from the Understanding Society COVID-19 study [19] covering ten months and three COVID-19 related national lockdown in the UK. Data was collected online monthly. The first wave of the COVID-19 survey was fielded between 24th-30th April 2020, with Wave 2 taking place in May, Wave 3 in June, Wave 4 in July, Wave 5 in September, Wave 6 in November and Wave 7 in December 2020/January 2021. There was no survey in August or October. The UK Household Longitudinal Study [20] (UKHLS; Understanding Society) is an ongoing panel survey of more than 40,000 households that began in 2009. Between 24-30th April 2020, members of households who participated in either of the two most recent UKHLS data collections (Waves 8 or 9), and who were older than 16 years, were invited to complete the first wave of the COVID-19 web survey. The probability sample was drawn from postal addresses. Northern Ireland and areas in England, Scotland, and Wales with proportionately large migrant and ethnic minority populations were oversampled. All household members aged 16 or older in April 2020 were invited to participate, except for those unable to make an informed decision as a result of incapacity, and those with unknown postal addresses or addresses abroad. The response rate (full interview) of seven waves of the Understanding Society COVID-19 study was 39%, 35%, 33%, 32%, 30%, 28% and 28% respectively [19].

For peer review only - http://bmjopen.bmj.com/site/about/guidelines.xhtml

The Understanding Society COVID-19 study is funded by the Economic and Social Research Council and the Health Foundation with scientific leadership by the Institute for Social and Economic Research, University of Essex. Fieldwork for the survey is carried out by Ipsos MORI and Kantar. The research data are distributed by the UK Data Service.

17,452 respondents took part in Wave 1 of the COVID-19 study. The inclusion criteria of the analytical sample for this study were all respondents aged 16 and over, who had participated in all seven waves survey and had no missing values on the outcome variable, constituting a final sample size of 8,163 individuals and 57,141 observations. The characteristics of the analytical sample are shown in Table 1. Compared with all participants in Wave 1, the analytical sample was slightly older and wealthier than the baseline sample. BAME individuals were less likely to continue to participate in the follow-up studies. Loss to follow-up was slightly more likely amongst those who reported sleep loss at wave 1 of the COVID-19 study.

Procedures

The outcome variables included whether the respondent reported an increase in sleep loss over worry in the last few weeks. The question on sleep loss was identical across both the Understanding Society COVID-19 Study and the mainstage data in 2019. As participants were not provided with a definition of sleep loss, it is recognised that different participants may have used different definitions when answering the question. The specific question wording, along with the four response categories, is presented in the text box below.

The next questions are about how you have been feeling over the last few weeks.

Have you recently lost much sleep over worry?
1. Not at all
2. No more than usual
3. Rather more than usual
4. Much more than usual

Source: University of Essex (2021).

In addition to the general sleep loss question, in the Wave 4 COVID-19 study, participants were asked a more detailed set of questions about sleep including: How many hours of actual sleep did you usually get per night during the last month? How often have you had trouble sleeping because you cannot get to sleep within 30 minutes? How often have you had trouble sleeping because you wake up in the middle of the night or early in the morning? These more comprehensive measures were compared against the general sleep loss measure as part of the descriptive analyses.

For the purposes of the multi-variate analysis, a respondent was defined as experiencing sleep loss during the lockdown if he or she reported sleep loss over worry in the last few weeks ‘rather more than usual’ or ‘much more than usual’ in the Understanding Society COVID-19 Study. The outcome variables were binary (1=yes; 0=no).

A range of explanatory variables were included, reflecting both known associates of sleep loss [9, 21] as well as those that we hypothesise may be associated with heightened anxiety during the pandemic.

For peer review only - http://bmjopen.bmj.com/site/about/guidelines.xhtml

Demographic and socioeconomic characteristics included age, gender, ethnicity and educational qualification. Gender distinguished between men and women; the survey responses do not differentiate those whose reported sex has changed since birth or those who classify themselves as intersex. Ethnicity was classified into three groups: British/English/Scottish/Welsh/Northern Irish (White), Other White, and Black, Asian, and Minority Ethnic (BAME).

Variables capturing factors associated with COVID-19 itself included whether the respondent reported having experienced symptoms that could be coronavirus and being a key worker [1,22]. Other variables aimed to capture the impacts of the policy response to COVID-19, particularly the effect of lockdown. Increased stress related to childcare and home schooling was proxied by the presence of children in the house (whether at least one child aged 0-4 or school-aged child), and whether the respondent was living with a partner. Exposure to financial stress was proxied by two variables capturing the respondents subjective view of their current and future financial situation. Social isolation was measured by the frequency of having felt lonely in the last 4 weeks. In order to capture regional differences in the intensity of the pandemic [2], a variable reflecting the respondents' place of respondence was included, based on the UK government office region (North East, North West, Yorkshire and The Humber, East Midlands, West Midlands, East of England, London, South East, South West, Wales, Scotland, and Northern Ireland). Finally, in order to capture time effects, we controlled for the survey time point, measured as a dummy variable (April, May, June, July, September, November and December/January).

Sleep loss and certain covariates such as the frequency of feelings of loneliness in the previous four weeks, has had symptoms that could be coronavirus, young children present in the household, and the respondents' perceived current and future financial situation, were measured at each wave of COVID-19 study. For some individuals, their responses to these questions changed over time during the follow-up period; thus, in the statistical models these were all treated as time-varying variables. Other covariates were treated as time-invariant variables. It is recognised that pregnancy, menstrual cycle and menopause may all be linked to sleep. Data on these was not available. However, age was included as a co-variant, which will partially capture whether women have reached the menopause.

Analytical approach

Descriptive statistics illustrating the prevalence of reported sleep loss (stratified by gender and ethnicity) pre and during the pandemic among *all* participants aged 16 and above in the USoc mainstage survey in 2019 and in the COVID-19 Study Waves 1 to 7 were calculated (Figure 1).

Subsequent analysis was then restricted to the analytical sample described above. Mixed-effects logistic models were used to assess the existence and strength of associations between sleep loss and COVID-19-related circumstances during the pandemic. Given that the data collected from an

[1] According to Department of Health and Social Care guidance on testing eligibility (2020) [22], key workers are people whose jobs are vital to public health and safety during the coronavirus lockdown. The list includes health and social care, e.g. all NHS staff, frontline health and social care staff such as doctors, nurses, plus support and specialist staff in the health and social care sector; education and childcare, including social workers; food and other necessary goods; key public services; local and national government; utility workers; public safety and national security; and transport.
[2] In the first few weeks of the pandemic in the UK, the rate of infection was much higher in London than elsewhere in the country.

For peer review only - http://bmjopen.bmj.com/site/about/guidelines.xhtml

individual over ten months are not independent of each other, mixed-effects models are the recommended statistical technique to take into consideration between-individuals variance and within-individuals variance [23]. This method has the advantage of including both fixed and random effects. The former are model components used to define systematic relationships such as overall changes of sleep loss over time and/or social determinants induced individual differences, and the latter account for the variability among individuals around the systematic relationships captured by the fixed effects.

For this study, the logistic mixed-effects models included sleep loss as the response variable, and gender, ethnicity group and timepoint as fixed effects. Observations by person-month were attached to each respondent; thus, in order to estimate the random intercepts, we accounted for random variation between individuals and between observations within the same individual. We modelled an interaction between time and gender, as well as between time and ethnicity, as a fixed effect to examine whether the association between gender and ethnicity varied according to time. Other potential confounding variables included the respondents' age, highest qualification, subjective current financial situation, subjective future financial situation, whether they were living with a partner, any children in the house, whether they were a key worker, the frequency of feelings of loneliness in the previous four weeks, any prior problem of sleep, and region. Regression analyses were carried out for the total population and then for men and women separately. Results were presented as odds ratios of sleep loss by gender and ethnicity, with 95% CIs and associated two-sided p values.

A sensitivity analysis was conducted in order to check the robustness of the results. Here we measured sleep loss as an ordered variable with four categories (not at all, no more than usual, rather more than usual and much more than usual) instead of two. Mixed effects ordered logistic regression was applied. The analyses were carried out in STATA version 15 [24].

Patient involvement

No patients were involved in this study.

Results

Descriptive analyses

Figure 1 shows the level of reported sleep loss by gender and ethnicity among all participants aged 16 and above in the USoc mainstage survey in 2019 and in the USoc: COVID-19 Study Wave 1 to Wave 7. The first data point provides a baseline, pre-pandemic, with subsequent data points then illustrating how sleep loss changed during the pandemic, with the first two national lockdowns highlighted, the third starting in January 2021. Black lines represent women in different ethnicities and grey lines represent men. Overall, women reported much higher levels of sleep loss than men; and respondents from ethnic minority groups had a higher prevalence of sleep loss than British White respondents among both men and women, with BAME women experiencing the highest prevalence. Differentials in the prevalence of reporting sleep loss by gender and ethnicity are evident at baseline; however, the gap appears to have widened during the pandemic and was especially pronounced in April 2020, illustrating the effect of the early stages of the pandemic and the first lockdown, when the prevalence of sleep loss rose amongst all groups, with the rise particularly

For peer review only - http://bmjopen.bmj.com/site/about/guidelines.xhtml

marked amongst all women and BAME men. As the first lockdown eased, the prevalence of sleep loss fell, reaching a low in July / September, although for most groups this remained slightly above the pre pandemic baseline. Sleep loss then increased during the autumn, with a second peak coinciding with the second national lockdown in November 2020. The prevalence of reported sleep loss pre and during the pandemic among the analytical sample (Appendix Figure 1) shows a similar pattern. However, sleep loss among BAME women was lower in the analytical sample than among all respondents without follow-up selection, whilst the level of sleep loss amongst "Other White" women was higher.

Restricting the analytical sample to all those with complete information across all seven waves, Table 2 shows that one in four people in the UK reported increased sleep loss due to worry during the first four weeks of the first coronavirus pandemic lockdown in spring 2020 (Table 2). There were clear differences between women and men and across ethnic communities. Strikingly, although men have been found to face a higher risk of experiencing severe symptoms and dying from COVID-19, women were twice as likely as men to report that they had lost sleep 'much more than usual' (5.8% v 1.9%) and 'rather more than usual' (23.9% v 11.8%), supporting the hypothesis that women were disproportionately affected by the economic and social consequences of the first lockdown. In the consecutive months, the proportion reporting increased sleep loss slightly decreased amongst both men and women, but women still showed a much higher proportion of increased sleep loss than men.

The results by ethnicity do, however, support the argument that the risk of infection may play a role; in the first month of lockdown in spring 2020, 6.3% of BAME respondents reported being 'much more than usual' to have lost sleep through worry over the last few weeks compared to 3.7% of white respondents (Table 2). Patterns of the changes of percent across waves are illustrated using stacked bar charts for gender and ethnicity in Figure 2a and Figure 2b.

The prevalence of sleep loss ('rather more than usual' and 'much more than usual') in April 2020 (22.0%) was higher than that reported prior to the pandemic (13.9%) and the differentials between men and women, and individuals from different ethnic communities have widened during the epidemic (Appendix Table A).

Looking at the more detailed information available in Wave 4 (July 2020) shows that on average, those who reported problems with sleep slept around 45 minutes less than those who did not have trouble sleeping (Table 3). The majority of respondents reporting sleep loss had trouble getting to sleep within 30 minutes or reported waking up in the middle of the night or early in the morning. They were also more likely to rate their overall quality of sleep fairly bad or very bad. They were also more likely to report having trouble sleeping because of having a cough or snoring loudly, taking medicine to help them sleep, and having trouble staying awake while driving, eating meals, or engaging in social activity (Table 3), indicating that the general sleep loss question is a good proxy for these wider set of sleep related issues.

Multi-variate analysis

Many of the characteristics associated with sleep loss are likely to be interrelated; for example, those with a child aged 0-4 at home are also likely to be aged 25-44; those who are key workers are more likely to have experienced symptoms etc. To further unravel the picture, a series of multivariate logistic regression models were run; firstly, for the population as a whole, and then separately for

For peer review only - http://bmjopen.bmj.com/site/about/guidelines.xhtml

men and women. The first two columns (A1 and A2) for Model A (Table 4) show the unadjusted (bi-variate) odds ratios of experiencing sleep loss by gender and ethnicity (OR=3.2, 95% CI=2.8–3.6; OR=1.4, 95% CI=1.6–2.4), whilst the third column (A3) shows the adjusted odds of the main effect for the full model.

The analysis shows that the differential reporting of sleep loss by gender remains significant even after controlling for all other factors (OR=2.1, 95% CI=1.9–2.4); the differential by ethnicity is however reversed (OR=0.7, 95% 0·5–0.8) once other factors are taken into account such as being a key worker, having had symptoms, having children in the household, experiencing financial difficulties and living in London, the initial epicentre of the UK's COVID-19 outbreak in spring 2020. Individuals from BAME communities are disproportionately represented in all of these groups [3] (Appendix Table B). By including interactions between gender and ethnicity with month, Model A4 assessed the effects of gender and ethnicity on shifts over time. The results show that the strength of the association between gender and ethnicity and the risk of sleep loss varied over time. Women presented a lower risk of sleep loss in May, June, July, November and January 2021 as compared to the early stages of the first lockdown in April 2020 (OR = 0.7, 95 % CI = 0.5 – 0.8; OR=0.6, 95% CI=0.5–0.8; OR=0.7, 95% CI=0.6–0.9; OR=0.8, 95% CI=0.6–0.9; OR=0.7, 95% CI=0.6–0.9). By contrast, the BAME group experienced a higher risk of sleep loss in May (OR=1.4, 95% CI=1.0–2.1), and the risk only started to fall in September (0.7 [0.5–1.0]).

The analysis shows that the coronavirus infection, with school-aged children at home, feeling lonely, perceived financial difficulties and worry, being a woman, and month were all predictive factors of sleep loss (Table 4). The influential factors were slightly different among men (Model B) and women (Model B). Among women, experiencing coronavirus symptoms was a risk factor, whilst being of BAME heritage reduced the risk of increased sleep loss *once other factors were controlled for*, whilst both these factors were not significant for men.

In the sensitivity analysis where sleep loss was measured as an ordered variable, the analysis of the data with mixed-effect ordered logistic regression rather than mixed-effect logistic regression did not change the pattern of results (Appendix Table C).

Discussion and conclusion

This study has revealed several important findings related to sleep health during the COVID-19 pandemic. Firstly it provides robust evidence that sleep loss is affecting more people during the COVID-19 pandemic than previously, reflecting the fact that stress levels have risen due to anxieties about health, financial consequences, changes in social life and the daily routine, all of which may affect sleep homeostasis.

The study also provides evidence that women have been more vulnerable to sleep deprivation during lockdown, which is in line with previous research suggesting that women have more sleep disturbances than men [9,21], and that women are more prone to stress-related sleep disorders such as

[3] A range of interaction effects with ethnicity were investigated but none were significant and thus are not included in the final model.

For peer review only - http://bmjopen.bmj.com/site/about/guidelines.xhtml

post-traumatic stress disorder and anxiety disorders [25]. There is emerging evidence that mental health experiences during the COVID-19 pandemic in the UK differ between men and women, with more women suffering from anxiety in the early stages of lockdown [26]. Women's position in the labour market may increase their exposure to COVID-19, as women represent a significant majority of frontline workers in social care, education and health care [16, 27]. Many parents will have been affected by school closures, and requirements to balance paid work with increasing childcare and providing support to their children's learning. However, the gendered allocation of childcare means that in many households, it is the mother who has continued to provide the majority of primary care for children. Furthermore, many mid-life women find themselves juggling employment with caring responsibilities for aged parents and grandchildren [28].

Individuals from BAME communities showed a higher prevalence and incidence rate of sleep loss than British White individuals. This reflects the fact that those of BAME heritage have disproportionally higher rates of coronavirus infection [14], high anxiety associated with coronavirus-specific circumstances, are more likely to be key workers, to have dependent children, and to feel lonely. All of these factors are likely to increase the risk of sleep loss, with the result that once all these other factors are controlled for, being a member of a BAME community was associated with a *reduced* chance of sleep loss – highlighting the complex relationship between ethnicity and sleep health. One possible hypothesised explanation might be that there are some differences in sleep patterns between the different ethnicities included in the BAME definition, given the vast social and cultural heterogeneity within the BAME population. Sleep is culturally practised across racial and ethnic groups [29]. For example, some cultures in Latin and Caribbean communities promote taking naps in the middle of the day (siesta) and biphasic sleep. Others endorse the polyphasic sleep modality in Asia and Africa, anchoring their sleep at night but taking several daytime naps as needed when under a social condition that allows sleep to occur [29]. A review study [30] shows that among the few published within racial/ethnic group analyses, there are differences in sleep between non-US-born and US-born racial/ethnic groups. Still, the group with the more favourable sleep profile is consistent for non-US-born Latinos compared to US-born Latinos and Whites but the pattern is unclear for other racial/ethnic minority groups. Future studies might investigate whether some ethnic minorities are more at risk of sleep loss to aid the design of more targeted sleep interventions.

Some limitations should be acknowledged. First, sleep loss was measured by self-reports and is therefore subject to recall bias and participants' perceptions [31]. Secondly, attrition between data collection waves and missing values on the outcome variable meant that follow-up data were not available for more than half of participants who were surveyed in Wave 1. The sub-sample lost to follow-up were older and less affluent than those who participated in subsequent waves. This attrition could have confounded the identification of prospective associations between gender, ethnicity and sleep loss, in that more vulnerable people were not retained in the analyses. Furthermore, loss to follow-up was slightly more likely amongst those who reported sleep loss at wave 1 of the COVID-19 study, which may lead to underestimated sleep loss in the study.

In conclusion, the COVID-19 pandemic and the policy responses to it, including home working and schooling, have widened the disparity of sleep deprivation across gender and ethnicity, putting women and ethnic minorities at an even greater disadvantage. Disrupted and poor sleep is associated with wider mental and physical health challenges. Policy makers and health professionals need to

For peer review only

For peer review only - http://bmjopen.bmj.com/site/about/guidelines.xhtml

take action now to support and promote better sleep health amongst vulnerable groups during the pandemic, averting future secondary complications.

For peer review only

For peer review only - http://bmjopen.bmj.com/site/about/guidelines.xhtml

Table 1. Analytical sample characteristics (Wave1).

	Analytical sample	Full wave 1 sample	P*
**Age, mean**	50.6 (SD=17.5) (8,163)	50.1 (SD=18.2) (17,452)	0.036
**Age group, % (n)**			<0.001
16-24	9.2 (309)	10.2 (1,543)	
25-44	27.3 (1,587)	28.6 (4,734)	
45-64	38.9 (3,571)	36.9 (7,028)	
65-74	16.6 (1,972)	15.0 (2,925)	
75+	8.0 (724)	9.4 (1,222)	
**Gender, % (n)**			0.092
Men	47.0 (3,418)	48.0 (7,287)	
Women	53.0 (4,745)	52.0 (10,165)	
**Ethnicity, % (n)**			<0.001
British/English/Scottish/Welsh/Northern Irish (White)	89.8 (7,138)	86.5 (14,029)	
Other White	3.3 (343)	3.7 (779)	
Black, Asian, and Minority Ethnic (BAME)	6.1 (577)	8.6 (2,044)	
**Highest qualification, % (n)**			0.001
No qualification	5.1 (317)	6.3 (650)	
GCSE or lower	28.3 (1,994)	29.9 (4,006)	
A level	22.8 (1,599)	22.4 (3,401)	
Degree	43.8 (4,210)	41.4 (8,195)	
**Live with a partner, % (n)**			0.041
No	35.3 (2,114)	36.1 (5,136)	
Yes	64.7 (6,049)	63.9 (12,316)	
**Children in the house, % (n)**			<.001
At least one child aged 0-4	8.2 (552)	9.0 (1,756)	
At least one school children aged 5-18	22.0 (1,488)	23.5 (4,410)	
No school children	69.8 (6,123)	67.4 (11,286)	
**Key worker, % (n)**			<.001
No	33.2 (2,479)	30.8 (5,588)	
Yes	27.1 (1,997)	25.1 (4,515)	
Not in paid or self-employed work	39.6 (3,687)	44.1 (7,349)	
**Has had symptoms that could be coronavirus, % (n)**			0.017
No	89.1 (7,266)	88.2 (15,250)	
Yes	10.9 (897)	11.8 (1,305)	
**Feel lonely, % (n)**			0.044
Hardly ever	62.3 (5,488)	59.1 (10,717)	
Sometime	28.9 (2,169)	29.3 (4,947)	
Often	8.8 (506)	8.4 (1,276)	
**Subjective current financial situation, % (n)**			<.001
Living comfortably	32.8 (3,261)	29.6 (5,815)	
Doing alright	42.2 (3,441)	40.1 (7,045)	
Just about getting by	19.6 (1,122)	17.2 (2,585)	
Finding it quite difficult/very difficult	5.4 (339)	6.2 (891)	
**Subjective future financial situation, % (n)**			<.001
Better off	6.5 (523)	7.1 (1,248)	
Worse off	16.0 (1,245)	15.3 (2,793)	
About the same	77.4 (6,395)	70.7 (12,278)	
**Prior problem of sleep, % (n)**			0.049
No	83.9 (7,031)	78.5 (13,668)	
Yes	16.1 (1,132)	15.8 (2,538)	
**Sleep loss, % (n)**			0.001
No	76.8 (6,371)	75.2 (12,008)	
Yes	23.2 (1,792)	24.8 (3,919)	
**Region, % (n)**			<.001
North East	4.8 (291)	4.2 (593)	
North West	9.9 (774)	10.9 (1,716)	
Yorkshire and The Humber	9.1 (712)	8.9 (1,482)	
East Midlands	8.7 (659)	7.7 (1,334)	
West Midlands	9.1 (669)	9.1 (1,479)	
East of England	9.8 (805)	10.0 (1,689)	
London	9.1 (658)	11.7 (1,849)	
South East	15.2 (1,208)	14.1 (2,428)	
South West	10.6 (880)	9.0 (1,598)	
Wales	4.3 (454)	4.5 (1,018)	
Scotland	7.0 (728)	7.6 (1,523)	
Northern Ireland	2.5 (325)	2.3 (742)	

Source: authors' analysis, Understanding Society: COVID-19 Study, 2020.
* p-value for comparison between participants in the analytical sample and all in the wave1.
Note: Proportions for the analytical sample are weighted using longitudinal weight; proportions for the entire sample at Wave 1 are weighted using cross-sectional weight. The number of respondents is unweighted. The mean age difference test used the T-test. Other categorical variables used the Pearson Chi-Square test.

For peer review only - http://bmjopen.bmj.com/site/about/guidelines.xhtml

Table 2. Prevalence of reported sleep loss at each wave of the Understanding Society COVID-19 Study (N=8,163).

		Not at all	No more than usual	Rather more than usual	Much more than usual
Wave 1 (April 2020)	All participants	35.3	42.4	18.3	4.0
	By gender	P<.001			
	Men	45.3	41.0	11.8	1.9
	Women	26.7	43.6	23.9	5.8
	By ethnicity	P<.001			
	British/English/Scottish/Welsh/Northern Irish (White)	35.5	42.3	18.5	3.7
	Other White	34.2	42.6	16.8	6.4
	Black, Asian, and Minority Ethnic (BAME)	18.5	16.8	16.7	6.3
Wave 2 (May 2020)	All participants	33.3	47.5	15.9	3.3
	By gender	P<.001			
	Men	42.6	43.4	11.8	2.2
	Women	24.9	51.2	19.7	4.2
	By ethnicity	P<.001			
	British/English/Scottish/Welsh/Northern Irish (White)	33.8	47.6	15.6	3.0
	Other White	30.2	40.6	23.8	5.4
	Black, Asian, and Minority Ethnic (BAME)	27.8	50.6	15.3	6.3
Wave 3 (June 2020)	All participants	30.1	49.6	16.5	3.8
	By gender	P<.001			
	Men	36.9	47.0	13.1	3.0
	Women	24.1	51.9	19.6	4.4
	By ethnicity	P<.001			
	British/English/Scottish/Welsh/Northern Irish (White)	30.7	49.4	16.4	3.5
	Other White	27.3	44.4	21.2	7.1
	Black, Asian, and Minority Ethnic (BAME)	24.3	55.2	13.9	6.5
Wave 4 (July 2020)	All participants	35.4	48.5	13.6	2.5
	By gender	P<.001			
	Men	44.9	43.6	10.2	1.3
	Women	26.9	52.8	16.6	3.7
	By ethnicity	P=.031			
	British/English/Scottish/Welsh/Northern Irish (White)	35.8	48.4	13.3	2.5
	Other White	30.1	53.1	15.3	1.5
	Black, Asian, and Minority Ethnic (BAME)	34.0	46.0	15.9	4.2
Wave 5 (September 2020)	All participants	31.0	52.3	13.8	2.9
	By gender	P<.001			
	Men	38.4	50.4	9.6	1.6
	Women	24.5	54.0	17.4	4.0
	By ethnicity	P=.004			
	British/English/Scottish/Welsh/Northern Irish (White)	31.3	52.1	13.7	2.9
	Other White	28.8	49.3	19.0	2.9
	Black, Asian, and Minority Ethnic (BAME)	31.5	53.6	12.5	2.3
Wave 6 (November 2020)	All participants	26.2	53.3	16.9	3.7
	By gender	P<.001			
	Men	33.6	51.4	12.8	2.2
	Women	19.7	54.9	20.5	5.0
	By ethnicity	P=.03			
	British/English/Scottish/Welsh/Northern Irish (White)	26.5	53.2	16.7	3.5
	Other White	22.5	53.2	20.6	3.7
	Black, Asian, and Minority Ethnic (BAME)	23.8	52.1	18.9	5.2
Wave 7 (January 2021)	All participants	27.5	53.0	15.9	3.7
	By gender	P<.001			
	Men	34.6	51.7	10.7	3.0
	Women	21.1	54.1	20.6	4.3
	By ethnicity	P<.001			
	British/English/Scottish/Welsh/Northern Irish (White)	28.2	52.3	16.1	3.5
	Other White	23.6	50.5	21.6	4.8
	Black, Asian, and Minority Ethnic (BAME)	20.1	64.2	11.0	4.8

Source: authors' analysis, Understanding Society: COVID-19 Study, 2020.
Note: All proportions are weighted using each wave cross-sectional individual web survey weight, beta version. Number of respondents are unweighted. Pearson Chi-Square test applied.

For peer review only - http://bmjopen.bmj.com/site/about/guidelines.xhtml

Table 3. Self-reported sleep loss and its association with more detailed information on trouble sleeping, July 2020.

	No sleep loss problem	Sleep loss problem	P value
Had trouble sleeping because cannot get to sleep within 30 minutes			P<0.001
Not during the past month	38.3	8.0	
Less than once a week	27.7	10.9	
Once or twice a week	20.6	25.3	
Three or more times a week	7.5	29.0	
More than once most nights	5.8	24.9	
Had trouble sleeping because wake up in the middle of the night or early in the morning			P<0.001
Not during the past month	28.5	2.2	
Less than once a week	23.9	8.8	
Once or twice a week	22.9	28.6	
Three or more times a week	13.4	31.4	
More than once most nights	10.8	28.8	
Had trouble sleeping because cough or snore loudly			P<0.001
Not during the past month	83.9	71.4	
Less than once a week	8.7	10.5	
Once or twice a week	4.2	9.5	
Three or more times a week	1.5	4.1	
More than once most nights	1.3	2.6	
Taken medicine to help sleep			P<0.001
Not during the past month	93.3	78.0	
Less than once a week	2.0	5.1	
Once or twice a week	1.8	5.1	
Three or more times a week	2.7	9.6	
Had trouble staying awake while driving, eating meals, or engaging in social activity			P<0.001
Not during the past month	89.7	71.8	
Less than once a week	6.0	13.3	
Once or twice a week	3.0	10.3	
Three or more times a week	1.1	2.5	
Overall quality of sleep			P<.001
Very good	20.1	0.6	
Fairly good	62.1	31.2	
Fairly bad	15.9	52.9	
Very bad	1.9	15.2	
Average sleep hours	7.09 (SD=1.22)	6.35 (SD=1.20)	P<.001
Number of respondents	6974	1189	

Source: authors' analysis, Understanding Society: COVID-19 Study, 2020. Wave4
Note: All proportions are weighted using wave4 cross-sectional individual web survey weight, beta version. Number of respondents are unweighted. Average sleep hours difference test used ANOVA F test, others used Pearson Chi-Square test.

For peer review only

For peer review only - http://bmjopen.bmj.com/site/about/guidelines.xhtml

Table 4. Multivariate modelling of the correlates of sleep loss during the pandemic (odds ratios and 95% CI)

	Model A All respondents				Model B Men		Model C Women	
	A1	A2	A3	A4	B1	B2	C1	C2
Gender								
Men (ref)								
Women	**3.2*** (2.8-3.6)**		**2.1*** (1.9-2.4)**	**2.8*** (2.4-3.3)**				
Ethnicity								
British/English/Scottish/Welsh/Northern Irish (White) (ref)								
Other White		**1.5** (1.2-2.1)**	1.1 (0.9-1.4)	0.8 (0.5-1.1)	1.3 (0.6-1.9)	0.8 (0.5-1.2)	**1.5* (1.1-2.1)**	**1.3ǂ (0.9-1.7)**
Black, Asian, and Minority Ethnic (BAME)		**1.4** (1.6-2.4)**	**0.7*** (0.5-0.8)**	**0.7* (0.5-0.9)**	**2.1*** (1.4-3.0)**	0.8 (0.6-1.1)	1.1 (0.9-1.5)	**0.6*** (0.5-0.8)**
Age group								
16-24 (ref)								
25-44			1.2 (0.9-1.6)	1.2 (0.9-1.6)		1.1 (0.6-2.1)		1.2 (0.9-1.7)
45-64			**1.3ǂ (0.9-1.7)**	**1.3ǂ (0.9-1.7)**		1.2 (0.7-2.2)		1.3 (0.9-1.8)
65-74			0.9 (0.7-1.2)	0.9 (0.7-1.2)		1.0 (0.5-1.9)		0.8 (0.6-1.2)
75+			**0.7* (0.5-0.9)**	**0.7* (0.5-0.9)**		**0.6ǂ (0.3-1.1)**		0.8 (0.5-1.2)
Highest qualification								
No qualification (ref)								
GCSE or lower			1.1 (0.9-1.3)	1.1 (0.9-1.3)		1.01 (0.8-1.5)		1.2 (0.9-1.4)
A level			**1.3** (1.1-1.7)**	**1.3** (1.1-1.7)**		**1.5ǂ (0.9-2.1)**		1.3 (0.9-1.7)
Degree			**1.4*** (1.2-1.7)**	**1.4*** (1.2-1.7)**		**1.5* (1.1-1.9)**		**1.4** (1.2-1.7)**
Subjective current financial situation								
Living comfortably (ref)								
Doing alright			**1.3*** (1.2-1.4)**	**1.3*** (1.2-1.4)**		**1.5*** (1.3-1.8)**		**1.2** (1.1-1.3)**
Just about getting by			**2.0*** (1.8-2.3)**	**2.0*** (1.8-2.3)**		**2.4*** (1.9-3.0)**		**1.9*** (1.6-2.2)**
Finding it quite difficult/very difficult			**3.8*** (3.1-4.6)**	**3.8*** (3.1-4.6)**		**4.3*** (3.0-6.1)**		**3.6*** (2.8-4.5)**
Subjective future financial situation								
Better off (ref)								
Worse off			**1.5*** (1.3-1.8)**	**1.5*** (1.3-1.8)**		**1.6*** (1.2-2.1)**		**1.5*** (1.2-1.7)**
About the same			0.9 (0.8-1.0)	0.9 (0.8-1.0)		0.9 (0.8-1.2)		0.9 (0.8-1.0)
Live with a partner								
No (ref)								
Yes			**1.2*** (1.1-1.4)**	**1.2*** (1.1-1.4)**		**1.3* (1.0-1.6)**		**1.2** (1.0-1.3)**
Children in the house								
None (ref)								
At least one child aged 0-4			1.1 (0.9-1.3)	1.1 (0.9-1.3)		1.1 (0.8-1.6)		1.1 (0.9-1.4)
At least one school children aged 5-18			**1.2** (1.0-1.3)**	**1.2** (1.0-1.3)**		1.2 (0.9-1.5)		**1.1ǂ (1.0-1.3)**
Key worker								
No (ref)								
Yes			1.0 (0.9-1.1)	1.0 (0.9-1.1)		0.9 (0.8-1.1)		1.1 (0.9-1.2)
Not in paid or self-employed work			**0.8** (0.7-0.9)**	**0.8** (0.7-0.9)**		**0.7** (0.6-1.0)**		0.9 (0.8-1.0)
Has had symptoms that could be coronavirus								
No (ref)								
Yes			**1.2** (1.1-1.4)**	**1.2** (1.1-1.4)**		1.2 (0.9-1.5)		**1.2* (1.0-1.4)**
Feel lonely								
Hardly ever (ref)								
Sometime			**3.4*** (3.2-3.7)**	**3.4*** (3.2-3.7)**		**4.3*** (3.7-5.0)**		**3.1*** (2.8-3.4)**
Often			**12.8*** (11.1-14.7)**	**12.8*** (11.1-14.7)**		**15.5*** (11.9-20.2)**		**11.7*** (9.9-13.8)**
Prior problem of sleep								
No (ref)								
Yes			**4.5*** (3.9-5.1)**	**4.5*** (3.9-5.1)**		**5.1*** (3.9-6.5)**		**4.1*** (3.5-4.8)**
Region								
North East (ref)								
North West			1.2 (0.9-1.6)	1.2 (0.9-1.6)		1.0 (0.6-1.8)		1.3 (0.9-1.9)
Yorkshire and The Humber			**1.3ǂ**	**1.3ǂ**		1.1		**1.5ǂ**

For peer review only - http://bmjopen.bmj.com/site/about/guidelines.xhtml

			**(0.9-1.9)**	**(0.9-1.9)**		(0.6-2.0)		**(1.0-2.2)**
East Midlands			1.2	1.2		1.2		1.3
			(0.9-1.7)	(0.9-1.7)		(0.7-2.1)		(0.9-1.9)
West Midlands			**1.4***	**1.4***		1.5		1.4
			**(1.0-1.9)**	**(1.0-1.9)**		(0.8-2.6)		(0.9-2.0)
East of England			**1.3ǂ**	**1.3ǂ**		1.5		1.2
			**(0.9-1.8)**	**(0.9-1.8)**		(0.9-2.7)		(0.8-1.8)
London			**1.7****	**1.7****		1.6		**1.7****
			**(1.2-2.4)**	**(1.2-2.4)**		(0.9-2.9)		**(1.2-2.6)**
South East			1.2	1.2		1.3		1.2
			(0.9-1.7)	(0.9-1.7)		(0.8-2.3)		(0.8-1.7)
South West			1.1	1.1		1.2		1.1
			(0.8-1.6)	(0.8-1.6)		(0.7-2.1)		(0.8-1.6)
Wales			1.2	1.2		1.3		1.1
			(0.8-1.6)	(0.8-1.6)		(0.7-2.3)		(0.7-1.7)
Scotland			1.2	1.2		1.2		1.2
			(0.9-1.7)	(0.9-1.7)		(0.7-2.1)		(0.8-1.8)
Northern Ireland			1.4	1.4		1.4		1.3
			(0.9-1.9)	(0.9-1.9)		(0.7-2.6)		(0.9-2.1)
Month								
April (ref)								
May			**0.7*****	**0.9**		0.9		**0.6*****
			**(0.6-0.8)**	**(0.8-1.1)**		(0.8-1.1)		**(0.6-0.7)**
June			**0.7*****	**1.0**		1.0		**0.7*****
			**(0.7-0.8)**	**(0.8-1.2)**		(0.8-1.2)		**(0.6-0.7)**
July			**0.5*****	**0.6*****		**0.6*****		**0.4*****
			**(0.4-0.5)**	**(0.5-0.7)**		**(0.5-0.7)**		**(0.4-0.5)**
September			**0.6*****	**0.7*****		**0.7*****		**0.6*****
			**(0.5-0.7)**	**(0.6-0.8)**		**(0.5-0.8)**		**(0.5-0.6)**
November			**0.7*****	**0.8ǂ**		**0.8ǂ**		**0.7*****
			**(0.6-0.8)**	**(0.7-1.0)**		**(0.7-1.0)**		**(0.6-0.8)**
January			**0.6*****	**0.8***		**0.8****		**0.6*****
			**(0.6-0.7)**	**(0.7-0.9)**		**(0.6-0.9)**		**(0.5-0.7)**
Interaction gender#month								
Women#May				**0.7*****				
				**(0.5-0.8)**				
Women#June				**0.6*****				
				**(0.5-0.8)**				
Women#July				**0.7****				
				**(0.6-0.9)**				
Women#September				0.8				
				(0.7-1.1)				
Women#November				**0.8***				
				**(0.6-0.9)**				
Women#January				**0.7****				
				**(0.6-0.9)**				
Interaction ethnicity#month								
BAME#May				**1.4ǂ**				
				**(1.0-2.1)**				
BAME#June				1.2				
				(0.8-1.7)				
BAME#July				1.1				
				(0.7-1.6)				
BAME#September				**0.7ǂ**				
				**(0.5-1.0)**				
BAME#November				1.0				
				(0.7-1.5)				
BAME#January				0.9				
				(0.6-3.4)				
Other White#May				**1.6***				
				**(1.0-2.7)**				
Other White#June				1.4				
				(0.9-2.2)				
Other White#July				1.2				
				(0.7-2.0)				
Other White#September				1.4				
				(0.9-2.3)				
Other White#November				1.5				
				(0.9-2.4)				
Other White#January				1.2				
				(0.7-1.9)				
LR test vs. logistic model (P value)	*<0.001*	*<0.001*	*<0.001*	*<0.001*	*<0.001*	*<0.001*	*<0.001*	*<0.001*
Number of person-month	*57,141*	*57,141*	*57,141*	*57,141*	*23,926*	*23,926*	*33,215*	*33,215*

Source: authors' analysis, Understanding Society: COVID-19 Study, 2020.
***p<0.001, **p<0.01, *p<0.05, ǂp<0.1

For peer review only - http://bmjopen.bmj.com/site/about/guidelines.xhtml

References

1.BBC News. Coronavirus: How the UK is sleeping under lockdown. [cited 2020 June 15]. Available: https://www.bbc.co.uk/news/health-52911395

2.Schwartz JRL, Roth T. Neurophysiology of sleep and wakefulness: basic science and clinical implications. Current Neuropharmacology 2008; **6**: 367–378.

3.Bertisch SM, Pollock BD, Mittleman MA, et al. Insomnia with objective short sleep duration and risk of incident cardiovascular disease and all-cause mortality: Sleep Heart Health Study. Sleep 2018; **41**: zsy047.

4.Liu TZ, Xu C, Rota M, et al. Sleep duration and risk of all-cause mortality: a flexible, non-linear, meta-regression of 40 prospective cohort studies. Sleep Medicine Reviews 2017; **32**: 28–36.

5.Taheri S. The link between short sleep duration and obesity: we should recommend more sleep to prevent obesity. Archives of Disease in Childhood 2006; **91**: 881–884.

6.Kline CE. The bidirectional relationship between exercise and sleep: implications for exercise adherence and sleep improvement. American Journal of Lifestyle Medicine 2014; **8**: 375–379.

7.Hafner M, Stepanek M, Taylor J, Troxel WM, van Stolk C. Why sleep matters—the economic costs of insufficient sleep: a cross-country comparative analysis. Rand Health Quarterly 2017; **6**: 11.

8.Perez-Pozuelo I, Zhai B, Palotti J, et al. The future of sleep health: a data-driven revolution in sleep science and medicine. npj Digital Medicine 2020; **3**: 42.

9.Nugent CN, Black LI. Sleep duration, quality of sleep, and use of sleep medication, by sex and family type, 2013–2014. NCHS Data Brief 2016; **230:** 1–8.

10.Vézina-Im L, Moreno JP, Thompson D, Nicklas TA, Baranowski T. Individual, social and environmental determinants of sleep among women: protocol for a systematic review and meta-analysis. BMJ Open 2017; **7:** e016592.

11.Grandner MA, Williams NJ, Knutson KL, Roberts D, Jean-Louis G. Sleep disparity, race/ethnicity, and socioeconomic position. Sleep Medicine 2016; **18:** 7–18.

12.Jackson CL, Redline S, Emmons KM. Sleep as a potential fundamental contributor to disparities in cardiovascular health. Annual Review of Public Health 2015; **36:** 417–40.

13.Hale L, Rivero-Fuentes E. Negative acculturation in sleep duration among Mexican immigrants and Mexican Americans. Journal of Immigrant Minor Health 2011; **13:** 402–7.

14.Public Health England. Disparities in the risk and outcomes of COVID-19. PHE publications, gateway number: GW-1311. [cited 2020 June 15]. Available: https://assets.publishing.service.gov.uk/government/uploads/system/uploads/attachment_data/file/892085/disparities_review.pdf

15.Evandrou M, Falkingham J, Feng Z, Vlachantoni A. Ethnic inequalities in limiting health and self-reported health in later life revisited. Journal of Epidemiology and Community Health 2016; **70:** 653-662.

For peer review only - http://bmjopen.bmj.com/site/about/guidelines.xhtml

16.Joyce R, Xu X. Shutdowns during the coronavirus crisis: which workers are most exposed? IFS Briefing Note BN279. [cited 2020 July 10]. Available: https://www.ifs.org.uk/uploads/BN278-Sector-Shutdowns.pdf

17.Andrew A, Cattan S, Dias MC, et al. How are mothers and fathers balancing work and family under lockdown? IFS Briefing Note BN290. [cited 2020 July 10]. Available: https://www.ifs.org.uk/uploads/BN290-Mothers-and-fathers-balancing-work-and-life-under-lockdown.pdf

18.BBC News 6th April 2020 'Coronavirus: Domestic abuse calls up 25% since lockdown, charity says' [cited 2020 July 10]. Available: https://www.bbc.co.uk/news/uk-52157620

19.University of Essex, Institute for Social and Economic Research. (2021). Understanding Society: COVID-19 Study, 2020-2021. [data collection]. 8th Edition. UK Data Service. SN: 8644, 10.5255/UKDA-SN-8644-8.

20.University of Essex, Institute for Social and Economic Research. Understanding Society: Waves 1-9, 2009-2018 and Harmonised BHPS: Waves 1-18, 1991-2009. [data collection]. 12th Edition. UK Data Service. 2020; SN: 6614, Available: http://doi.org/10.5255/UKDA-SN-6614-13

21.Grandner MA, Jackson NJ, Izci-Balserak B, et al. Social and behavioral determinants of perceived insufficient sleep. Frontiers in Neurology 2015; **6**: 112.

22.Department of Health and Social Care. Guidance Coronavirus (COVID-19): getting tested. [cited 2020 June 15]. Available: https://www.gov.uk/guidance/coronavirus-covid-19-getting-tested.

23.Bell1 A, Fairbrother M, Jones K. Fixed and random effects models: making an informed choice. Quality & Quantity 2018 https://doi.org/10.1007/s11135-018-0802-x.

24.StataCorp. Stata Statistical Software: Release 15. 2017.College Station, TX: StataCorp LLC.

25.Li SH, Graham BM. Why are women so vulnerable to anxiety, trauma-related and stress-related disorders? The potential role of sex hormones. Lancet Psychiatry 2017; **4:** 73–82.

26.Office for National Statistics (ONS). Coronavirus and anxiety, Great Britain: 3 April 2020 to 10 May 2020. [cited 2020 June 15]. Available: https://www.ons.gov.uk/peoplepopulationandcommunity/wellbeing/articles/coronavirusandanxietygreatbritain/3april2020to10may2020

27.Office for National Statistics (ONS). Coronavirus and key workers in the UK. [cited 2020 June 15]. Available: https://www.ons.gov.uk/employmentandlabourmarket/peopleinwork/earningsandworkinghours/articles/coronavirusandkeyworkersintheuk/2020-05-15

28.Gomez-leon M, Evandrou M, Falkingham J, Vlachantoni A. The dynamics of social care and employment in mid-life. Ageing & Society 2019; **39**: 381-408.

For peer review only - http://bmjopen.bmj.com/site/about/guidelines.xhtml

29. Williams NJ, Grandner MA, Snipes SA, Rogers A, Williams O, Airhihenbuwa C, Jean-Louis G. Racial/ethnic disparities in sleep health and health care: importance of the sociocultural context. Sleep Health 2015; 1: 28–35.

30. Johnson DA, Jackson CL, Williams NJ, Alcántara C. Are sleep patterns influenced by race/ethnicity – a marker of relative advantage or disadvantage? Evidence to date. Nature and Science of Sleep 2019; 11:79–95.

31.Lauderdale DS, Knutson KL, Yan LL, Liu K, Rathouz PJ. Sleep duration: how well do self-reports reflect objective measures? The CARDIA Sleep Study. Epidemiology 2008; **19:** 838–845. doi: 10.1097/EDE.0b013e318187a7b0

Figure 1. Prevalence of reported sleep loss pre and during the pandemic among all participants aged 16 and above in Understanding Society mainstage survey in 2019 and Understanding Society: COVID-19 Study Wave 1 to Wave 7.

Figure 2. Prevalence of reported sleep loss at each wave of the Understanding Society COVID-19 Study (N=8,163).

Appendix Figure 1. Prevalence of reported sleep loss pre and during the pandemic among analytical sample in Understanding Society mainstage survey in 2019 and Understanding Society: COVID-19 Study Wave 1 to Wave 7.

For peer review only

Figure 1. Prevalence of reported sleep loss pre and during the pandemic among all participants aged 16 and above in Understanding Society mainstage survey in 2019 and Understanding Society: COVID-19 Study Wave 1 to Wave 7.

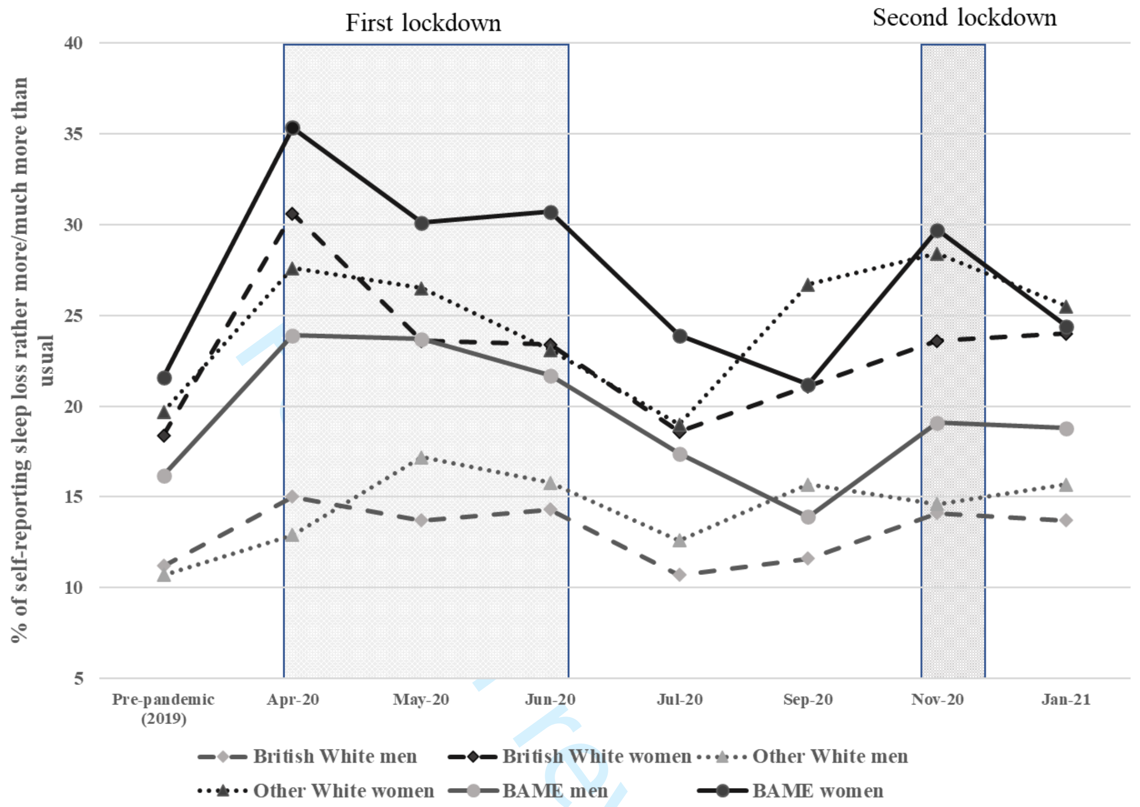

Source: authors' analysis, Understanding Society: COVID-19 Study, 2020.
Note: All proportions are unweighted. Black lines represent women in different ethnicities and grey lines represent men.
Number of respondents: N=29,685 (Pre-pandemic 2019), N=15,668 (2020 April), N=14,154 (2020 May), N=13,437 (2020 June), N=13,075 (2020 July), N= 12,170 (2020 September), N=11,472 (2020 November), N=11,299 (2021 January).

Figure 2. Prevalence of reported sleep loss at each wave of the Understanding Society COVID-19 Study (N=8,163).

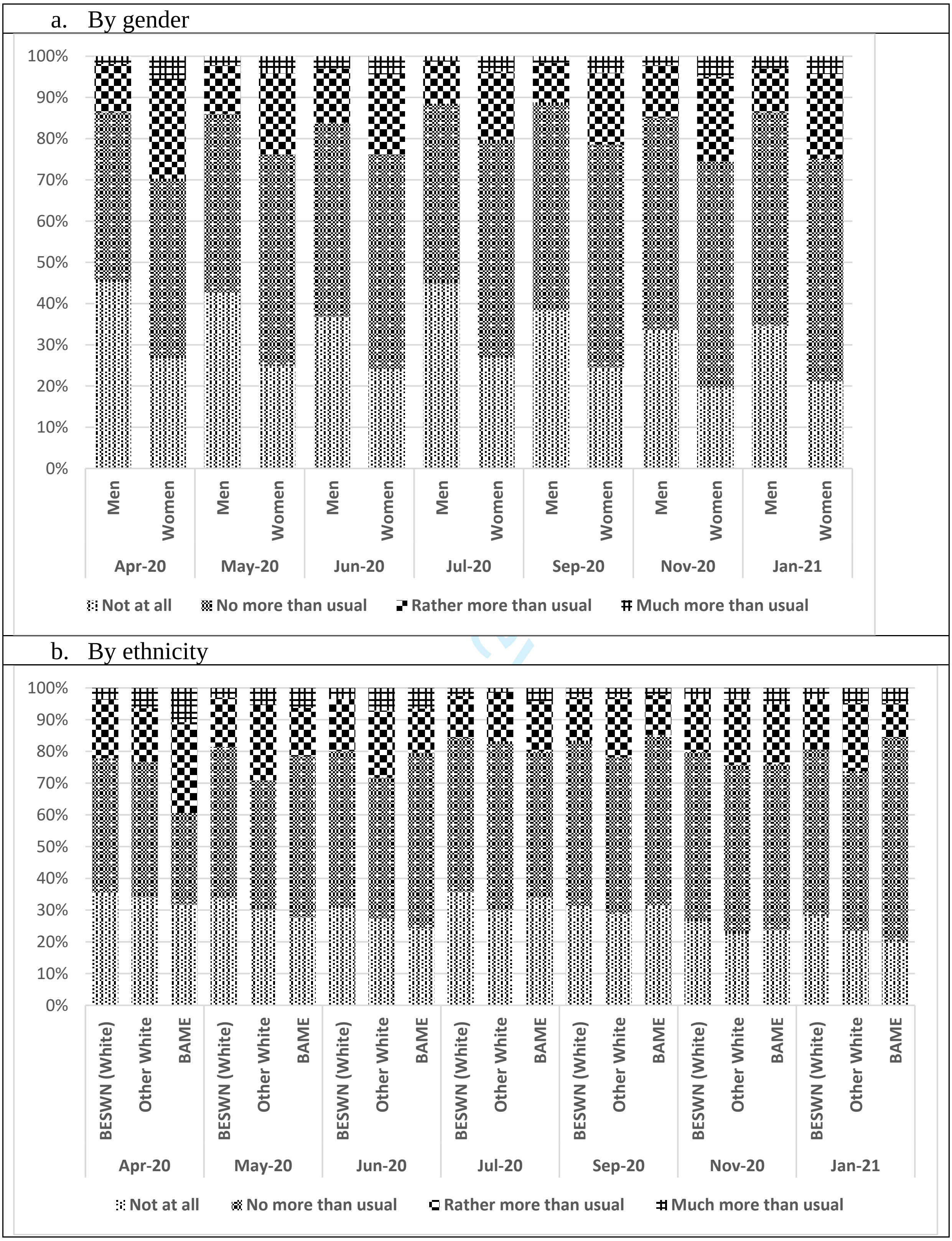

Source: authors' analysis, Understanding Society: COVID-19 Study, 2020.
Note: All proportions are weighted using each wave cross-sectional individual web survey weight, beta version. BESWN: British/English/Scottish/Welsh/Northern Irish.

For peer review only - http://bmjopen.bmj.com/site/about/guidelines.xhtml

Appendix Table A. Sleep loss comparison before and after the COVID-19 pandemic by sex and ethnicity (N=8,163).

	Sleep loss before the COVID-19 pandemic (2019)	Sleep loss after the COVID-19 pandemic (April 2020)
Total	13.9	22.0
Sex	P<0.001	P<0.001
Men	10.2	13.5
Women	16.5	28.0
Ethnicity	P=0.05	P=0.438
British/English/Scottish/Welsh/Northern Irish (White)	13.5	21.7
Other White	15.5	23.3
Black, Asian, and Minority Ethnic (BAME)	17.2	24.3

Source: authors' analysis, Wave 1 of Understanding Society: COVID-19 Study, 2020.

Note: All proportions and number of respondents are unweighted. Pearson Chi-Square test applied.

For peer review only

Appendix Table B. Characteristics of participants, by ethnicity (N=8,163).

	British/English/Scottish/Welsh/Northern Irish (White)	Other White	Black, Asian, and Minority Ethnic (BAME)
Age group **% (n)**	P<0.001		
16-24	4.6 (199)	1.0 (1)	12.2 (54)
25-44	21.1 (1,286)	26.1 (76)	30.4 (194)
45-64	41.5 (3,114)	50.2 (174)	43.7 (268)
65-74	22.7 (1,850)	16.7 (72)	10.4 (48)
75+	10.1 (689)	5.9 (20)	3.3 (13)
Gender **% (n)**	P=0.111		
Men	46.2 (3,009)	38.6 (122)	47.0 (244)
Women	53.8 (4,129)	61.4 (221)	53.0 (333)
Subjective current financial situation **% (n)**	P<0.001		
Living comfortably	36.8 (2,949)	33.7 (127)	21.9 (149)
Doing alright	42.5 (3,016)	42.1 (147)	40.4 (228)
Just about getting by	16.1 (915)	19.3 (54)	28.1 (140)
Finding it quite difficult/very difficult	4.6 (258)	5.0 (15)	9.6 (60)
Subjective future financial situation **% (n)**	P<0.001		
About the same	79.3 (5,640)	66.8 (249)	68.9 (420)
Better off	5.8 (450)	10.4 (28)	6.7 (37)
Worse off	14.9 (1,048)	22.8 (66)	24.4 (120)
Children in the house **% (n)**	P<0.001		
No school aged child	76.3 (5,503)	68.5 (239)	60.0 (314)
At least one child aged 0-4	6.3 (449)	5.4 (24)	11.5 (72)
At least one school children aged 5-18	17.3 (1,186)	26.1 (80)	28.5 (191)
Key worker **% (n)**	P<0.001		
No	30.4 (2,130)	35.1 (121)	29.6 (189)
Yes	24.9 (1,709)	30.2 (87)	28.1 (177)
Not in paid or self-employed work	44.7 (3,299)	34.7 (135)	42.2 (211)
Has had symptoms that could be coronavirus **% (n)**	P=0.012		
No	89.8 (6,375)	90.6 (308)	83.7 (489)
Yes	10.2 (763)	9.4 (35)	16.3 (88)
Feel lonely **% (n)**	P<0.001		
Hardly ever	65.5 (4,889)	64.4 (228)	58.3 (329)
Sometime	27.0 (1,821)	26.2 (94)	33.2 (203)
Often	7.4 (428)	9.4 (21)	8.5 (45)
Prior sleep loss **% (n)**	P=0.557		
No	84.9 (6,176)	82.7 (290)	87.0 (478)
Yes	15.1 (962)	17.3 (53)	13.0 (99)
Region **% (n)**	P<0.001		
North East (ref)	5.0 (278)	1.5 (4)	0.7 (4)
North West	10.3 (684)	5.9 (20)	8.9 (62)
Yorkshire and The Humber	9.4 (633)	5.4 (19)	7.8 (54)
East Midlands	8.5 (611)	4.5 (14)	4.4 (30)
West Midlands	8.8 (563)	3.5 (21)	10.0 (76)
East of England	10.5 (725)	9.9 (28)	4.4 (41)
London	7.1 (373)	21.8 (65)	40.4 (210)
South East	15.7 (1,094)	12.4 (46)	11.5 (58)
South West	11.4 (820)	9.4 (30)	6.7 (20)
Wales	4.4 (423)	5.0 (16)	1.9 (12)
Scotland	7.5 (693)	5.4 (15)	3.3 (8)
Northern Ireland	1.6 (241)	15.3 (65)	0.1 (2)

Source: authors' analysis, Understanding Society: COVID-19 Study, 2020.

Note: All proportions are weighted using sample weights. The percentages are 100% within each ethnic group. Number of respondents are unweighted. Pearson Chi-Square test applied.

For peer review only

Appendix Table C. Sensitivity analyses: odds ratios and 95% CI of mixed-effects ordered logistic regressions.

	Model A All respondents			
	A1	A2	A3	A4
Gender				
Men (ref)				
Women	**3.6*** (3.2-4.0)**		**2.5*** (2.2-2.7)**	**3.4*** (3.0-3.9)**
Ethnicity				
British/English/Scottish/Welsh/Northern Irish (White) (ref)				
Other White		**1.6** (1.2-2.1)**	1.1 (0.9-1.4)	0.9 (0.7-1.2)
Black, Asian, and Minority Ethnic (BAME)		**1.6*** (1.3-2.1)**	0.9 (0.7-1.1)	**0.8* (0.6-0.9)**
Month				
April (ref)				
May			0.9 (0.9-1.0)	**1.1* (1.0-1.3)**
June			**1.1** (1.0-1.2)**	**1.4*** (1.3-1.6)**
July			**0.7*** (0.7-0.8)**	**0.8*** (0.5-0.6)**
September			**0.9 (0.9-1.0)**	**1.2** (1.1-1.4)**
November			**1.2*** (1.1-1.3)**	**1.5*** (1.3-1.6)**
January			1.1 (0.9-1.1)	**1.3*** (1.2-1.5)**
Interaction gender#month				
Women#May				**0.7*** (0.6-0.8)**
Women#June				**0.6*** (0.5-0.7)**
Women#July				**0.7*** (0.6-0.8)**
Women#September				**0.7*** (0.6-0.8)**
Women#November				**0.7*** (0.6-0.8)**
Women#January				**0.7*** (0.6-0.8)**
Interaction ethnicity#month				
BAME#May				**1.3* (1.0-1.7)**
BAME#June				1.2 (0.9-1.5)
BAME#July				**1.3* (1.0-1.7)**
BAME#September				0.9 (0.7-1.2)
BAME#November				1.1 (0.8-1.4)
BAME#January				1.2 (0.9-1.6)
Other White#May				1.3 (0.9-1.8)
Other White#June				1.2 (0.9-1.7)
Other White#July				1.0 (0.7-1.5)
Other White#September				1.2 (0.9-1.8)
Other White#November				1.2 (0.9-1.8)
Other White#January				1.1 (0.8-1.6)
LR test vs. ologit model (P value)	*<0.001*	*<0.001*	*<0.001*	*<0.001*
Number of person-month	*57,141*	*57,141*	*57,141*	*57,141*

Source: authors' analysis, Understanding Society: COVID-19 Study, 2020.
***p<0.001, **p<0.01, *p<0.05
Notes: Model A1 and A2 were bivariate models with gender or ethnicity in the model. Model A3 and A4 were multivariate models, controlling for age, highest qualification, subjective current financial situation, subjective future financial situation, live with a partner, children in the house, key worker, felt lonely, prior problem of sleep, region and month.

For peer review only

Appendix Figure 1. Prevalence of reported sleep loss pre and during the pandemic among analytical sample in Understanding Society mainstage survey in 2019 and Understanding Society: COVID-19 Study Wave 1 to Wave 7.

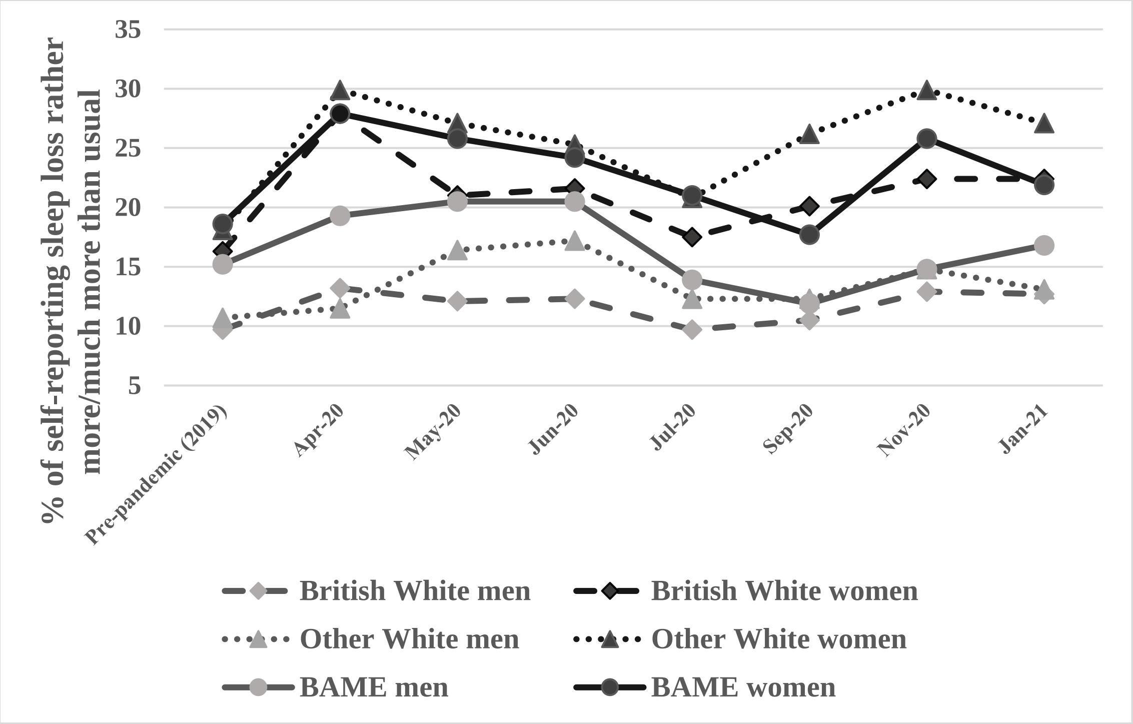

Source: authors' analysis, Understanding Society: COVID-19 Study, 2020.
Note: All proportions are unweighted. Black lines represent women in different ethnicities and grey lines represent men.
Number of respondents: N=8,163.

STROBE 2007 (v4) Statement—Checklist of items that should be included in reports of *cohort studies*

Section/Topic	Item #	Recommendation	Reported on page #
**Title and abstract**	1	(*a*) Indicate the study's design with a commonly used term in the title or the abstract	1,2
		(*b*) Provide in the abstract an informative and balanced summary of what was done and what was found	2
**Introduction**			
Background/rationale	2	Explain the scientific background and rationale for the investigation being reported	4-5
Objectives	3	State specific objectives, including any prespecified hypotheses	5
**Methods**			
Study design	4	Present key elements of study design early in the paper	5
Setting	5	Describe the setting, locations, and relevant dates, including periods of recruitment, exposure, follow-up, and data collection	5-7
Participants	6	(*a*) Give the eligibility criteria, and the sources and methods of selection of participants. Describe methods of follow-up	6
		(*b*) For matched studies, give matching criteria and number of exposed and unexposed	-
Variables	7	Clearly define all outcomes, exposures, predictors, potential confounders, and effect modifiers. Give diagnostic criteria, if applicable	6-7
Data sources/ measurement	8*	For each variable of interest, give sources of data and details of methods of assessment (measurement). Describe comparability of assessment methods if there is more than one group	
Bias	9	Describe any efforts to address potential sources of bias	6
Study size	10	Explain how the study size was arrived at	6
Quantitative variables	11	Explain how quantitative variables were handled in the analyses. If applicable, describe which groupings were chosen and why	6-7
Statistical methods	12	(*a*) Describe all statistical methods, including those used to control for confounding	7-8
		(*b*) Describe any methods used to examine subgroups and interactions	7-8
		(*c*) Explain how missing data were addressed	7-8
		(*d*) If applicable, explain how loss to follow-up was addressed	6
		(*e*) Describe any sensitivity analyses	8
**Results**			

For peer review only

Participants	13*	(a) Report numbers of individuals at each stage of study—eg numbers potentially eligible, examined for eligibility, confirmed eligible, included in the study, completing follow-up, and analysed	6,8
		(b) Give reasons for non-participation at each stage	6,8
		(c) Consider use of a flow diagram	-
Descriptive data	14*	(a) Give characteristics of study participants (eg demographic, clinical, social) and information on exposures and potential confounders	8-9
		(b) Indicate number of participants with missing data for each variable of interest	6
		(c) Summarise follow-up time (eg, average and total amount)	8
Outcome data	15*	Report numbers of outcome events or summary measures over time	8-9
Main results	16	(*a*) Give unadjusted estimates and, if applicable, confounder-adjusted estimates and their precision (eg, 95% confidence interval). Make clear which confounders were adjusted for and why they were included	9-10
		(*b*) Report category boundaries when continuous variables were categorized	-
		(*c*) If relevant, consider translating estimates of relative risk into absolute risk for a meaningful time period	-
Other analyses	17	Report other analyses done—eg analyses of subgroups and interactions, and sensitivity analyses	10
**Discussion**			
Key results	18	Summarise key results with reference to study objectives	10
**Limitations**			
Interpretation	20	Give a cautious overall interpretation of results considering objectives, limitations, multiplicity of analyses, results from similar studies, and other relevant evidence	10-11
Generalisability	21	Discuss the generalisability (external validity) of the study results	11
**Other information**			
Funding	22	Give the source of funding and the role of the funders for the present study and, if applicable, for the original study on which the present article is based	3

*Give information separately for cases and controls in case-control studies and, if applicable, for exposed and unexposed groups in cohort and cross-sectional studies.

Note: An Explanation and Elaboration article discusses each checklist item and gives methodological background and published examples of transparent reporting. The STROBE checklist is best used in conjunction with this article (freely available on the Web sites of PLoS Medicine at http://www.plosmedicine.org/, Annals of Internal Medicine at http://www.annals.org/, and Epidemiology at http://www.epidem.com/). Information on the STROBE Initiative is available at www.strobe-statement.org.

For peer review only
